# Supplementary figures and images for: Lipid hydroperoxides promote sarcopenia through carbonyl stress
Source: eLife. 2023 Mar 23;12:e85289. doi: 10.7554/eLife.85289 (PMC10076018; doi:10.7554/eLife.85289)

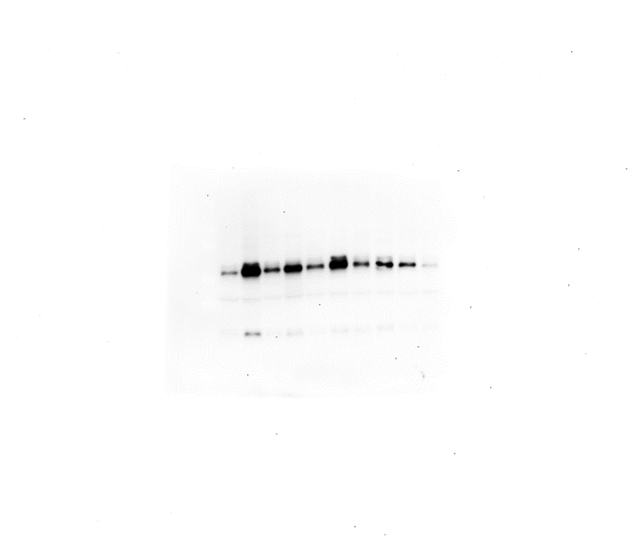

Supplement: Figure 1—source data 1. [file elife-85289-fig1-data1.zip › Figure 1-Source Data 1/Fig 1E 4HNE.png]

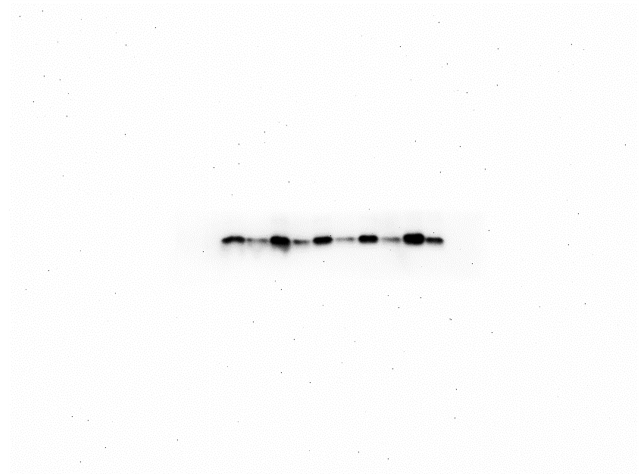

Supplement: Figure 1—source data 1. [file elife-85289-fig1-data1.zip › Figure 1-Source Data 1/Fig 1E GPx4.png]

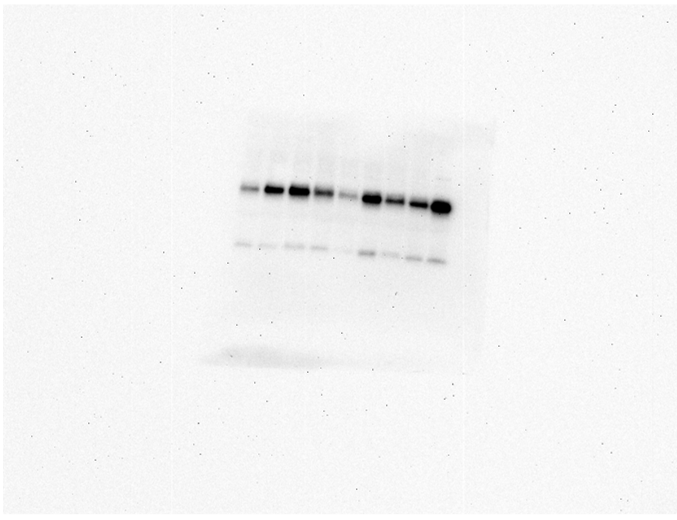

Supplement: Figure 1—figure supplement 1—source data 1. [file elife-85289-fig1-figsupp1-data1.zip › Figure 1-supplemental figure 1 - Source Data 1/Figure 1S1F 4HNE.png]

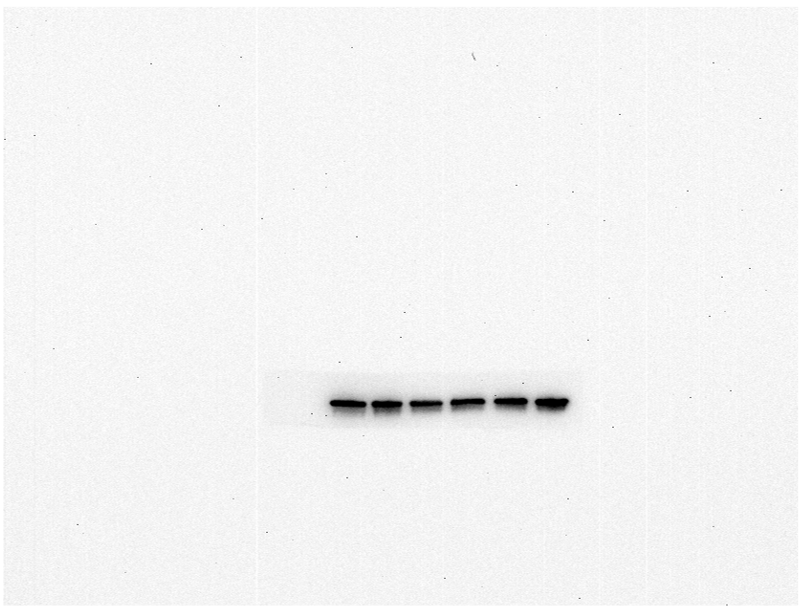

Supplement: Figure 1—figure supplement 1—source data 1. [file elife-85289-fig1-figsupp1-data1.zip › Figure 1-supplemental figure 1 - Source Data 1/Figure 1S1F GAPDH.png]

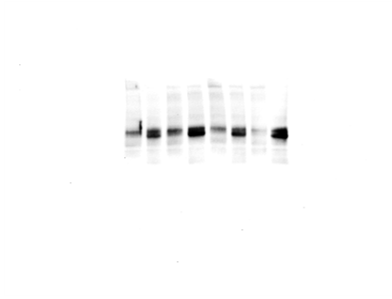

Supplement: Figure 2—source data 1. [file elife-85289-fig2-data1.zip › Figure 2-Source Data 1/Figure 2E 4HNE.png]

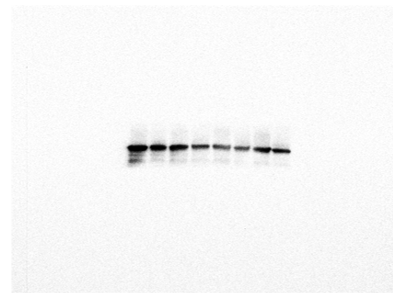

Supplement: Figure 2—source data 1. [file elife-85289-fig2-data1.zip › Figure 2-Source Data 1/Figure 2E Actin.png]

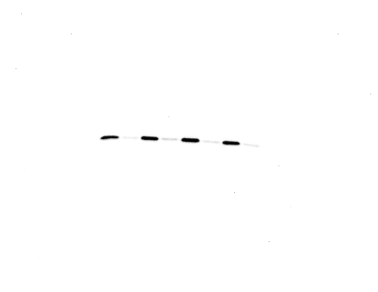

Supplement: Figure 2—source data 1. [file elife-85289-fig2-data1.zip › Figure 2-Source Data 1/Figure 2E GPx4.png]

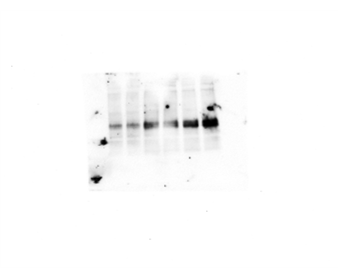

Supplement: Figure 2—figure supplement 1—source data 1. [file elife-85289-fig2-figsupp1-data1.zip › Figure 2-supplemental figure 1 - Source Data 1/Figure 2S1B 4HNE.png]

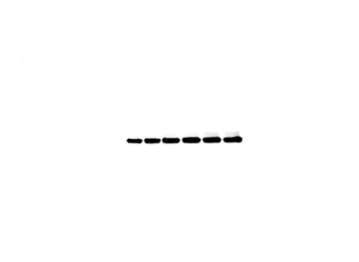

Supplement: Figure 2—figure supplement 1—source data 1. [file elife-85289-fig2-figsupp1-data1.zip › Figure 2-supplemental figure 1 - Source Data 1/Figure 2S1B Actin.png]

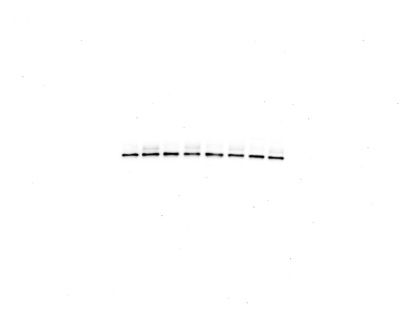

Supplement: Figure 2—figure supplement 1—source data 1. [file elife-85289-fig2-figsupp1-data1.zip › Figure 2-supplemental figure 1 - Source Data 1/Figure 2S1G Actin.png]

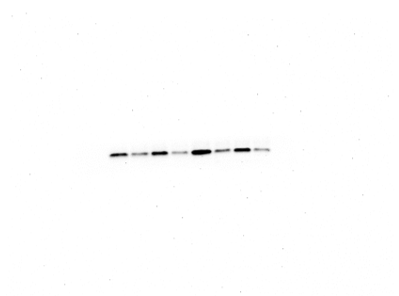

Supplement: Figure 2—figure supplement 1—source data 1. [file elife-85289-fig2-figsupp1-data1.zip › Figure 2-supplemental figure 1 - Source Data 1/Figure 2S1G GPx4.png]

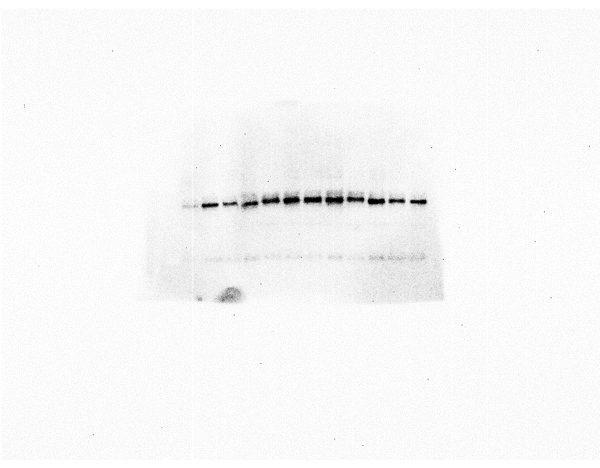

Supplement: Figure 3—source data 1. [file elife-85289-fig3-data1.zip › Figure 3-Source Data 1/Figure 3B 4HNE.png]

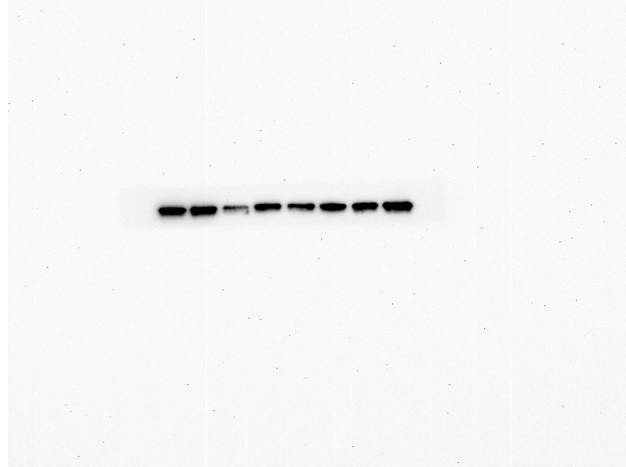

Supplement: Figure 3—source data 1. [file elife-85289-fig3-data1.zip › Figure 3-Source Data 1/Figure 3B GAPDH.png]

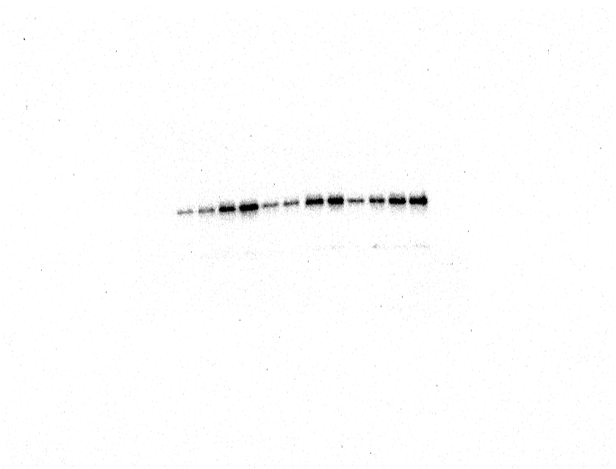

Supplement: Figure 3—source data 1. [file elife-85289-fig3-data1.zip › Figure 3-Source Data 1/Figure 3G 4HNE.png]

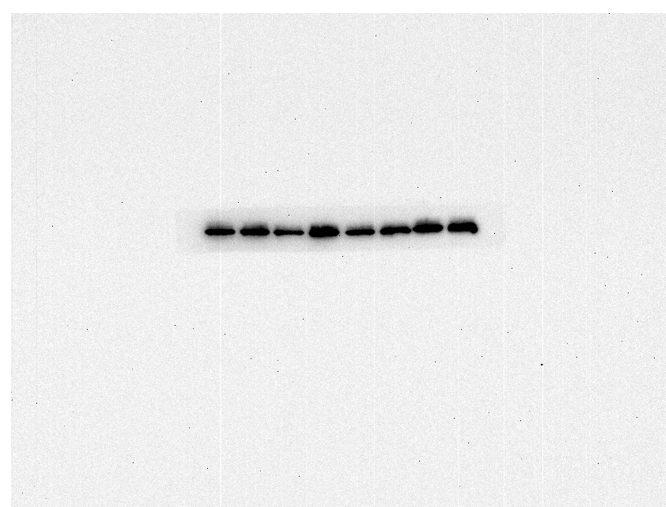

Supplement: Figure 3—source data 1. [file elife-85289-fig3-data1.zip › Figure 3-Source Data 1/Figure 3G GAPDH.png]

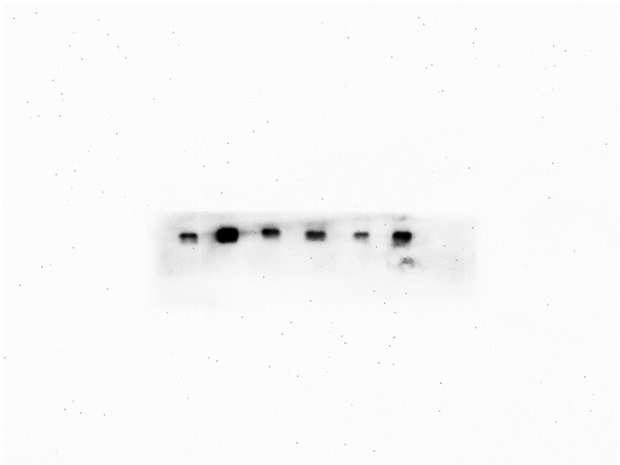

Supplement: Figure 3—source data 1. [file elife-85289-fig3-data1.zip › Figure 3-Source Data 1/Figure 3G GPX4.png]

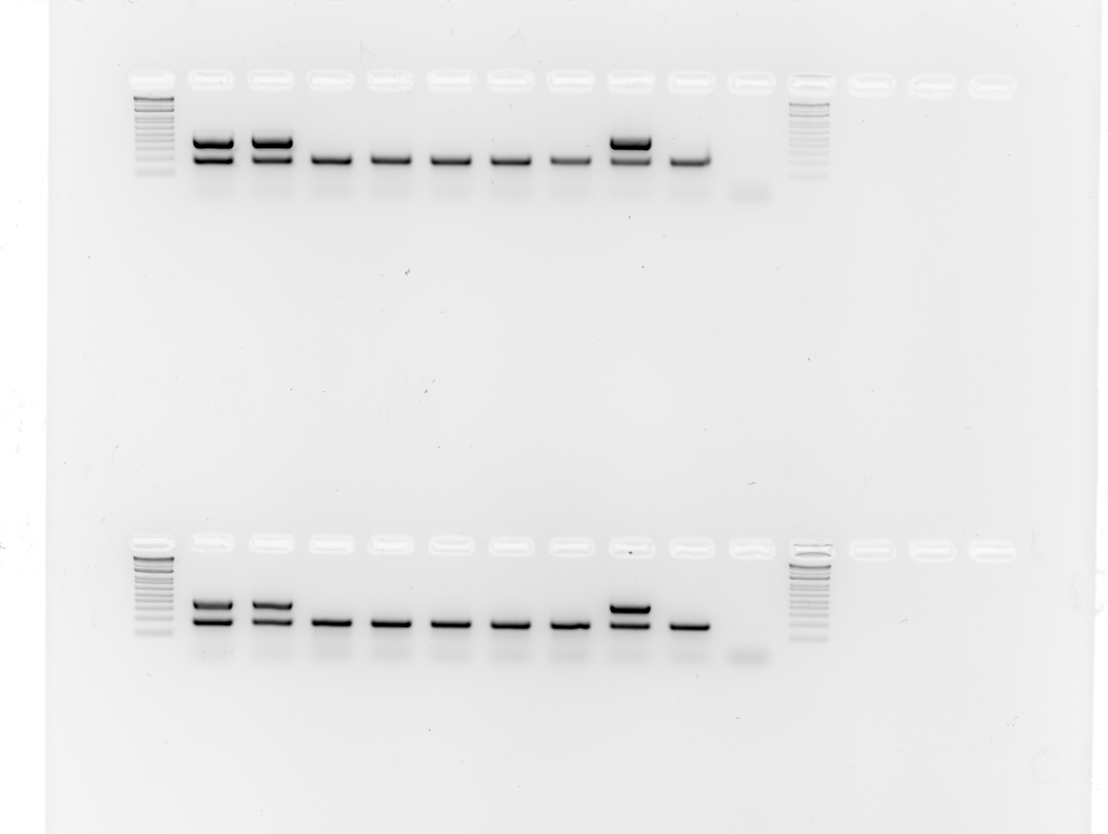

Supplement: Figure 3—figure supplement 2—source data 1. [file elife-85289-fig3-figsupp2-data1.zip › Figure 3-supplemental figure 2 - Source Data 1/Figure 3S2A Cre.png]

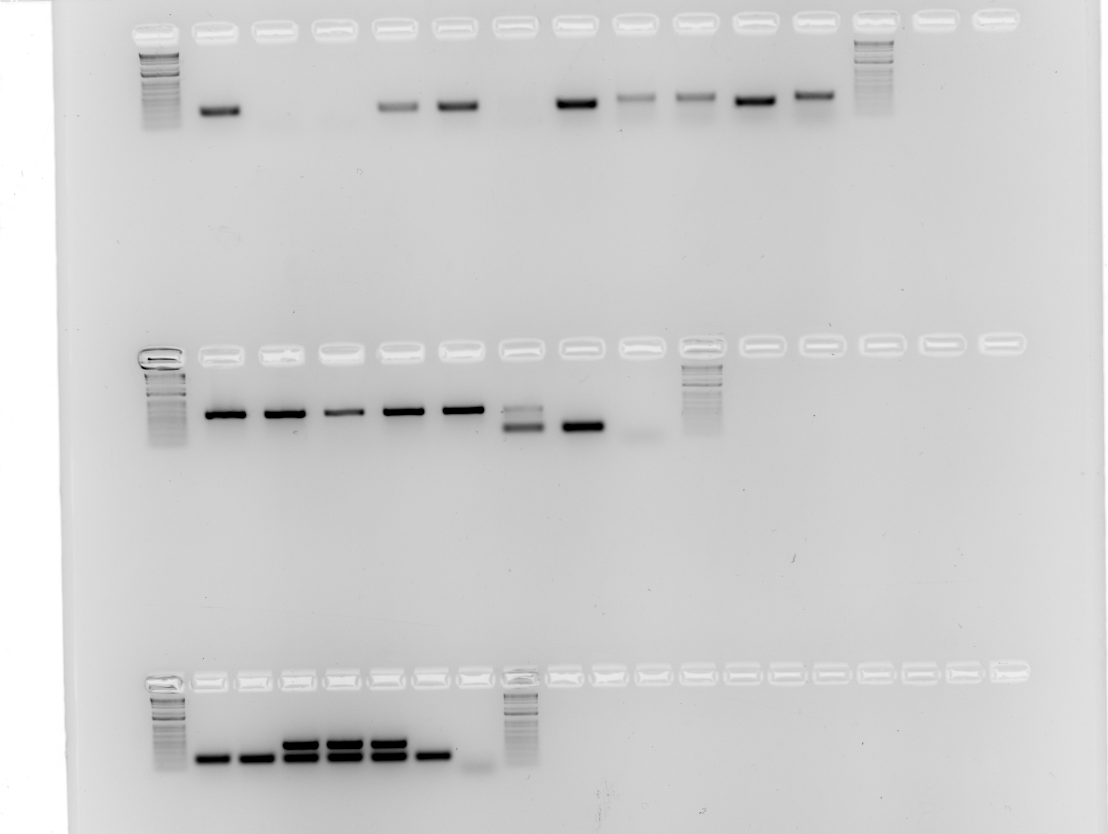

Supplement: Figure 3—figure supplement 2—source data 1. [file elife-85289-fig3-figsupp2-data1.zip › Figure 3-supplemental figure 2 - Source Data 1/Figure 3S2A GPx4 LoxP.png]

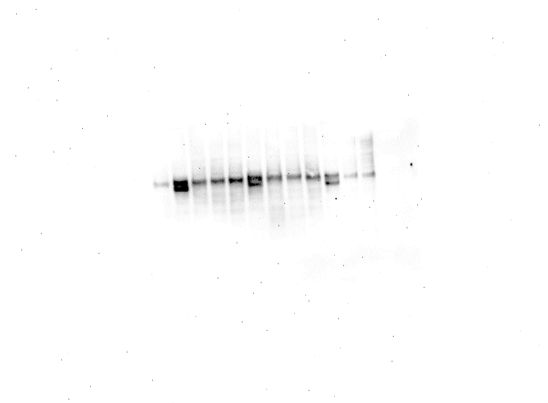

Supplement: Figure 4—source data 1. [file elife-85289-fig4-data1.zip › Figure 4-Source Data 1/Figure 4B 4HNE.png]

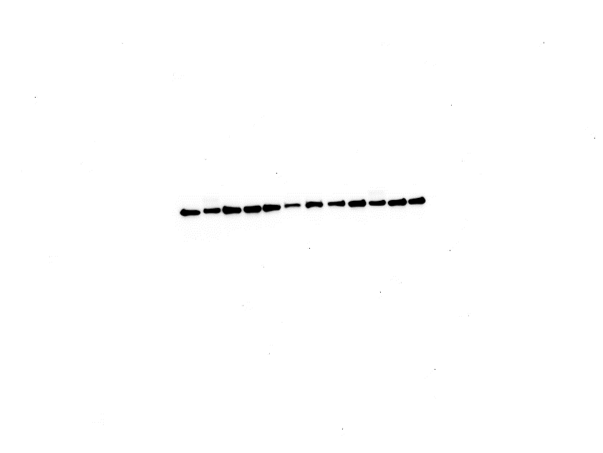

Supplement: Figure 4—source data 1. [file elife-85289-fig4-data1.zip › Figure 4-Source Data 1/Figure 4B Actin.png]

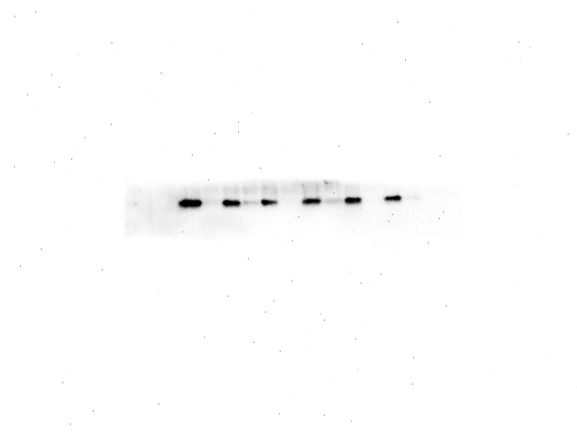

Supplement: Figure 4—source data 1. [file elife-85289-fig4-data1.zip › Figure 4-Source Data 1/Figure 4B GPX4.png]

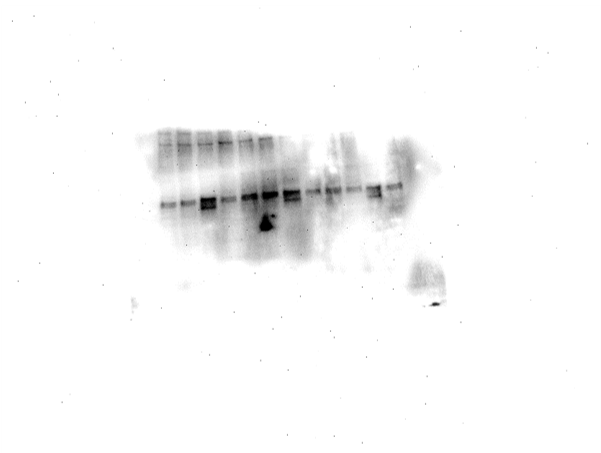

Supplement: Figure 4—source data 1. [file elife-85289-fig4-data1.zip › Figure 4-Source Data 1/Figure 4F 4HNE.png]

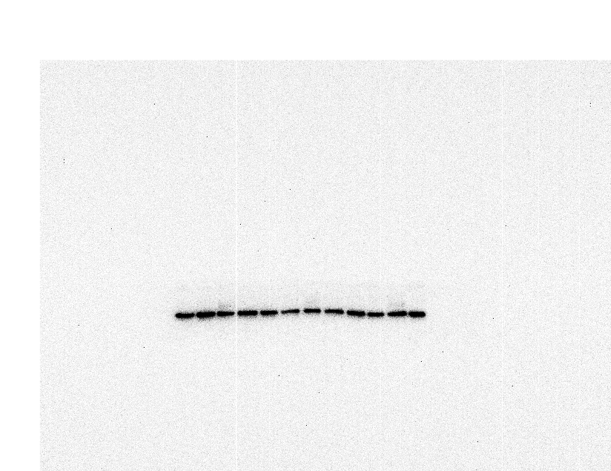

Supplement: Figure 4—source data 1. [file elife-85289-fig4-data1.zip › Figure 4-Source Data 1/Figure 4F Actin.png]

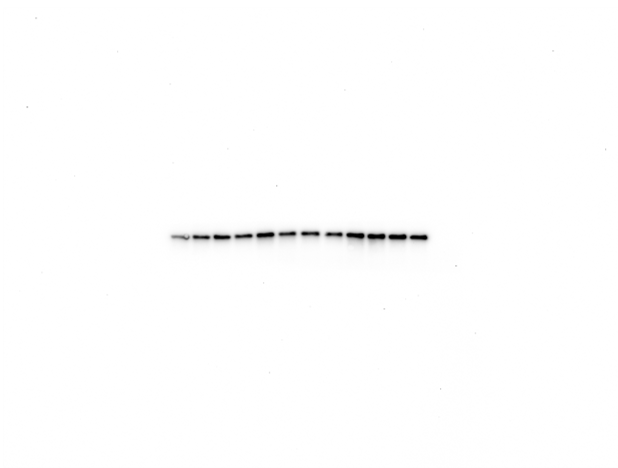

Supplement: Figure 5—source data 1. [file elife-85289-fig5-data1.zip › Figure 5-Source Data 1/Figure 5C Actin.png]

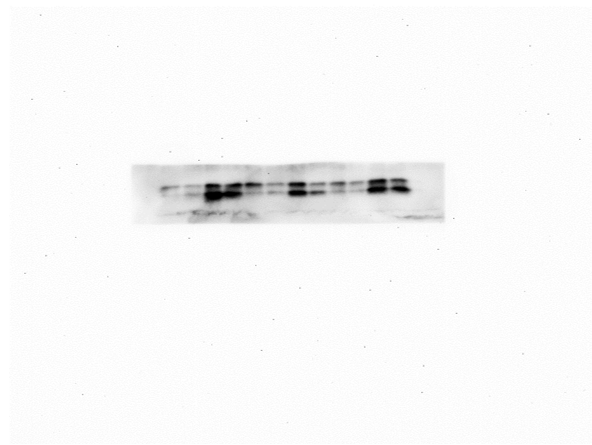

Supplement: Figure 5—source data 1. [file elife-85289-fig5-data1.zip › Figure 5-Source Data 1/Figure 5C LC3.png]

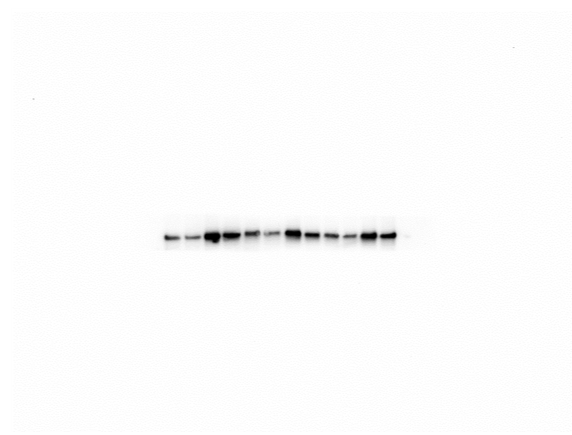

Supplement: Figure 5—source data 1. [file elife-85289-fig5-data1.zip › Figure 5-Source Data 1/Figure 5C p62.png]

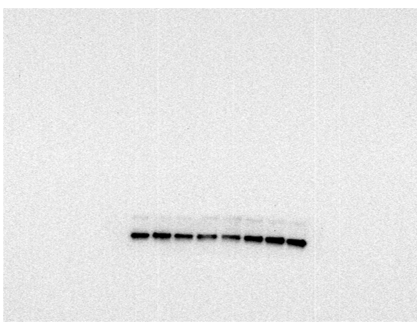

Supplement: Figure 5—figure supplement 1—source data 1. [file elife-85289-fig5-figsupp1-data1.zip › Figure 5-supplemental figure 1 - Source Data 1/Figure 5S1D Actin.png]

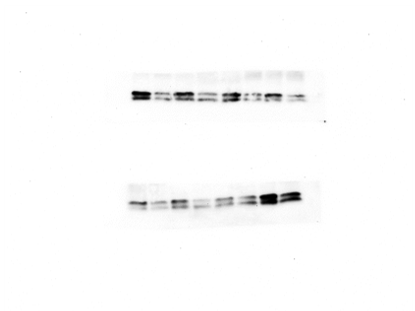

Supplement: Figure 5—figure supplement 1—source data 1. [file elife-85289-fig5-figsupp1-data1.zip › Figure 5-supplemental figure 1 - Source Data 1/Figure 5S1D LC3.png]

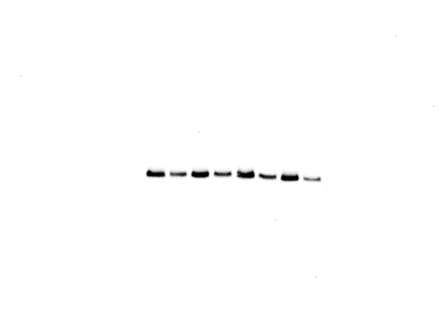

Supplement: Figure 5—figure supplement 1—source data 1. [file elife-85289-fig5-figsupp1-data1.zip › Figure 5-supplemental figure 1 - Source Data 1/Figure 5S1D p62.png]

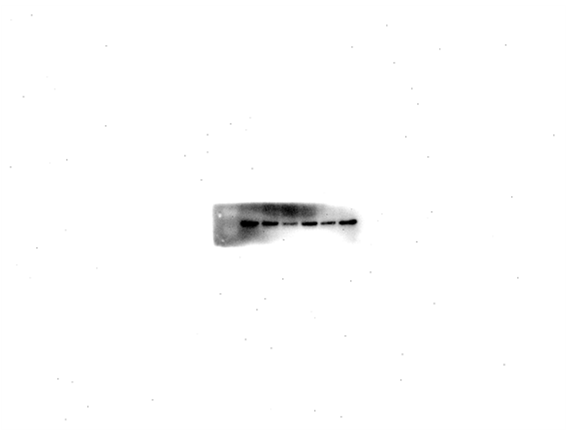

Supplement: Figure 5—figure supplement 1—source data 1. [file elife-85289-fig5-figsupp1-data1.zip › Figure 5-supplemental figure 1 - Source Data 1/Figure 5S1K Actin.png]

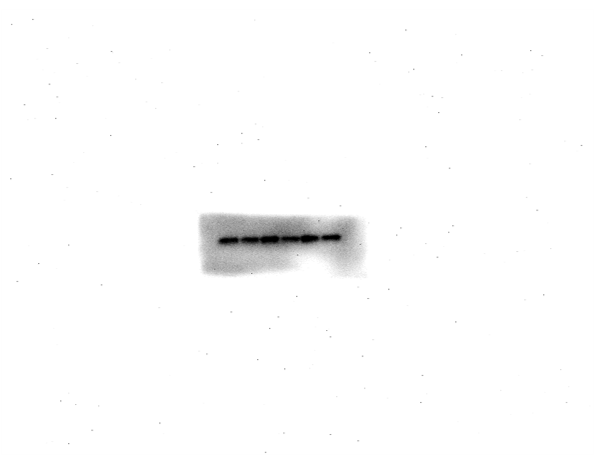

Supplement: Figure 5—figure supplement 1—source data 1. [file elife-85289-fig5-figsupp1-data1.zip › Figure 5-supplemental figure 1 - Source Data 1/Figure 5S1K GPx4.png]

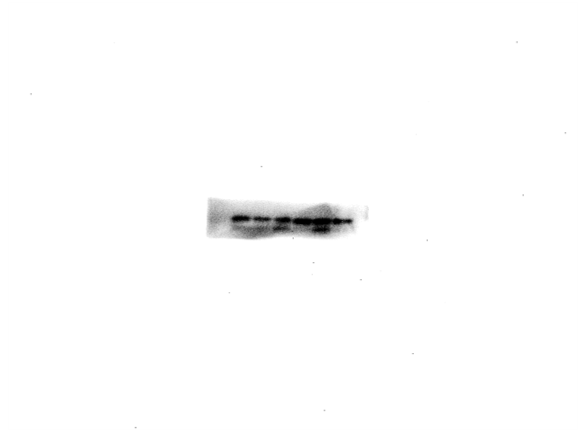

Supplement: Figure 5—figure supplement 1—source data 1. [file elife-85289-fig5-figsupp1-data1.zip › Figure 5-supplemental figure 1 - Source Data 1/Figure 5S1K LC3.png]

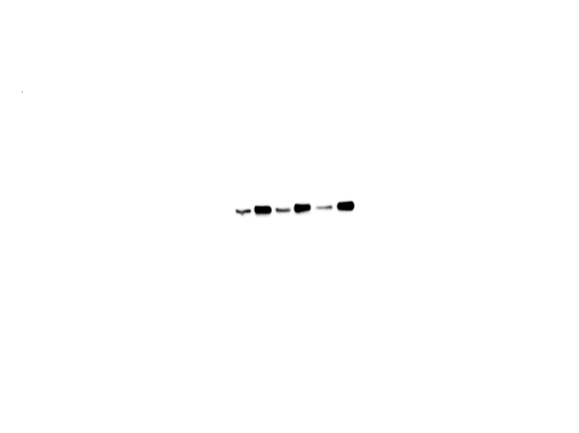

Supplement: Figure 5—figure supplement 1—source data 1. [file elife-85289-fig5-figsupp1-data1.zip › Figure 5-supplemental figure 1 - Source Data 1/Figure 5S1K p62.png]

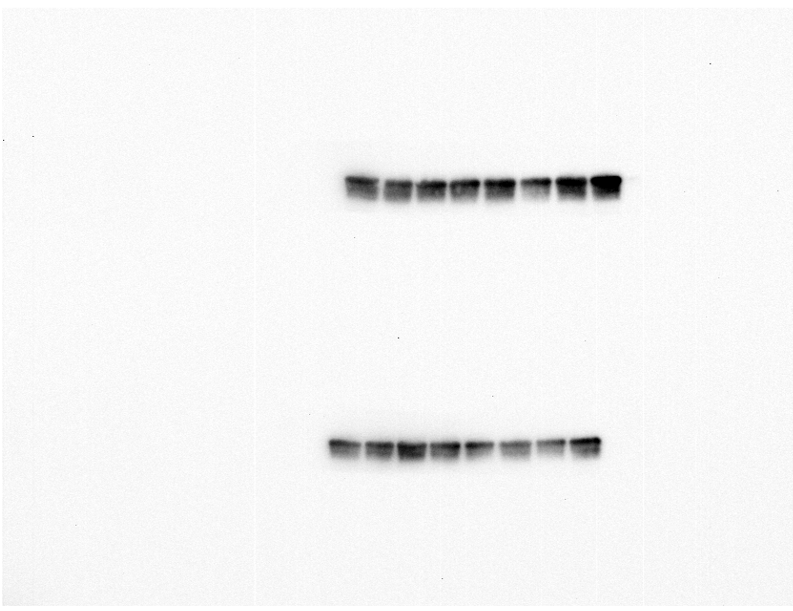

Supplement: Figure 6—source data 1. [file elife-85289-fig6-data1.zip › Figure 6-Source Data 1/Figure 6B Continued GAPDH 2.png]

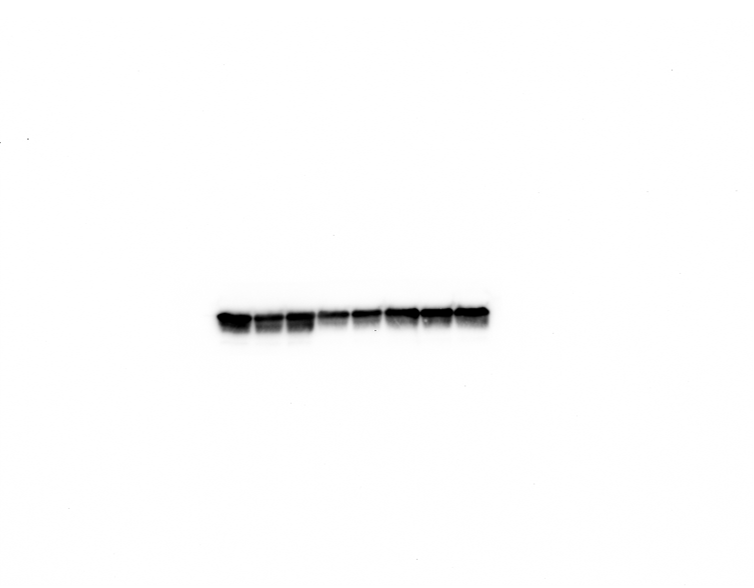

Supplement: Figure 6—source data 1. [file elife-85289-fig6-data1.zip › Figure 6-Source Data 1/Figure 6B Continued GAPDH.png]

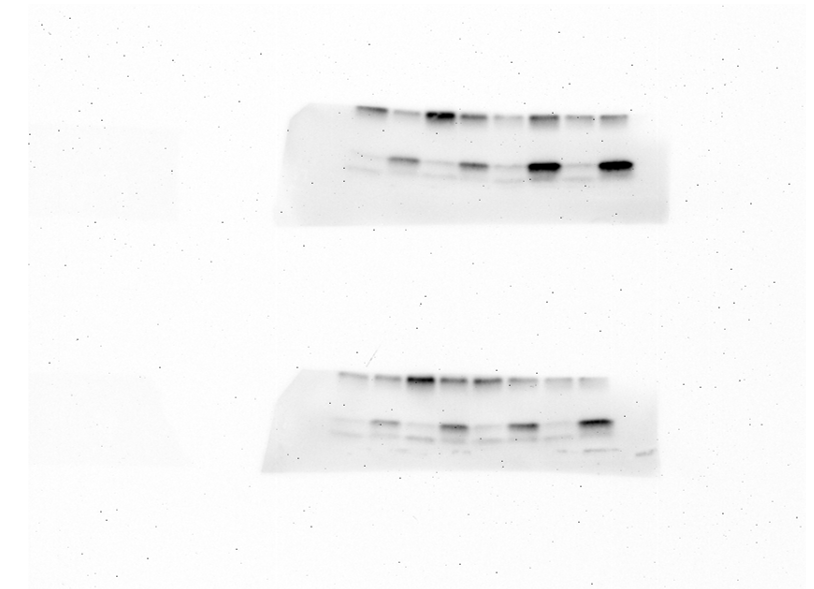

Supplement: Figure 6—source data 1. [file elife-85289-fig6-data1.zip › Figure 6-Source Data 1/Figure 6B Continued LC3.png]

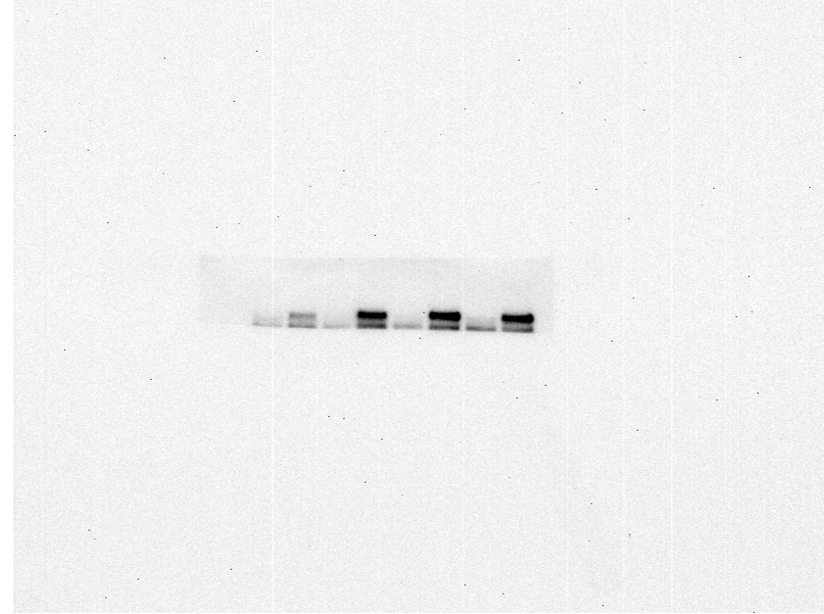

Supplement: Figure 6—source data 1. [file elife-85289-fig6-data1.zip › Figure 6-Source Data 1/Figure 6B Continued p62 2.png]

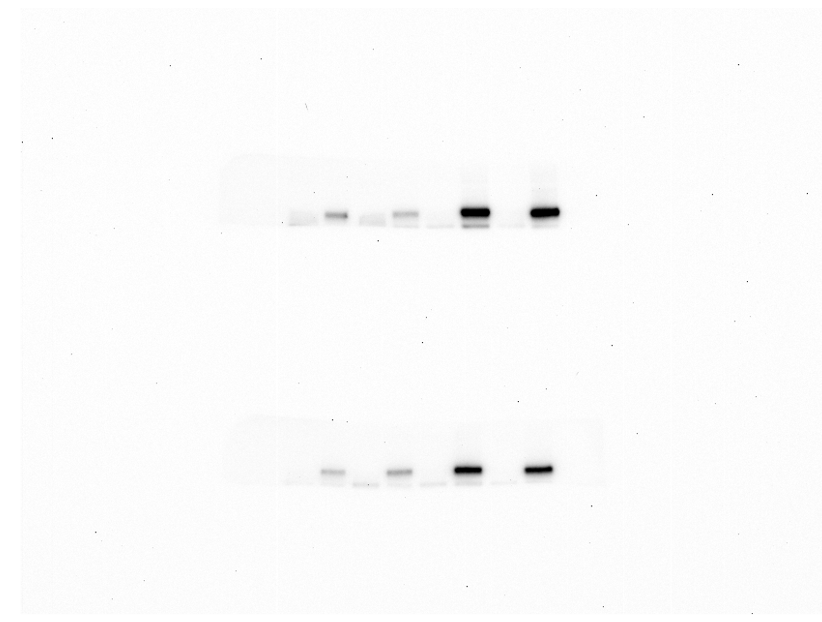

Supplement: Figure 6—source data 1. [file elife-85289-fig6-data1.zip › Figure 6-Source Data 1/Figure 6B Continued p62.png]

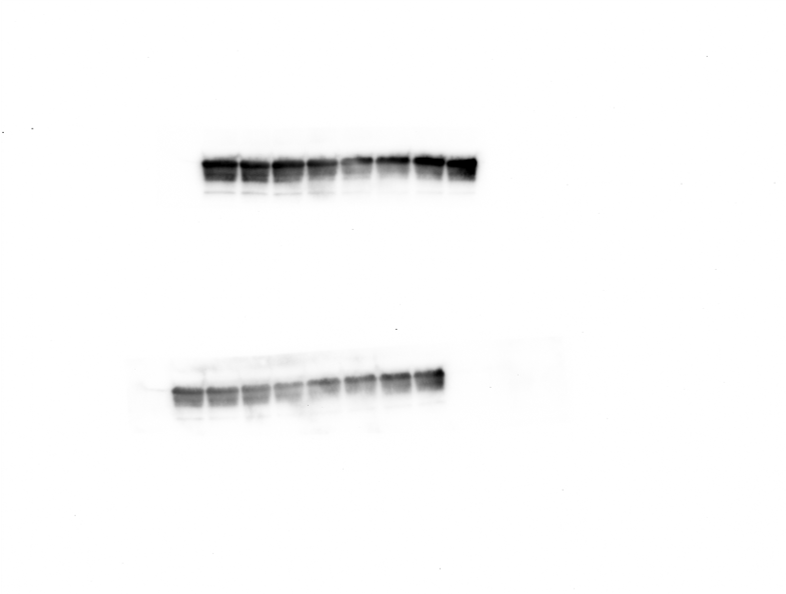

Supplement: Figure 6—source data 1. [file elife-85289-fig6-data1.zip › Figure 6-Source Data 1/Figure 6B GAPDH.png]

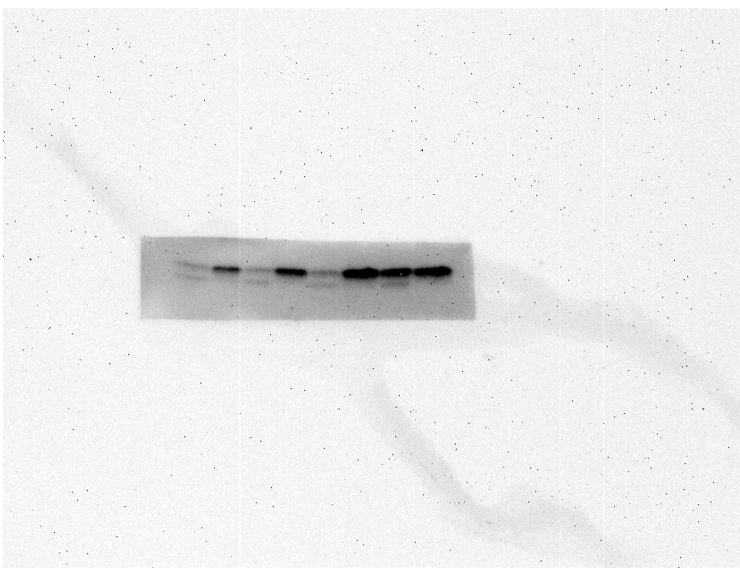

Supplement: Figure 6—source data 1. [file elife-85289-fig6-data1.zip › Figure 6-Source Data 1/Figure 6B LC3 Continued 2.png]

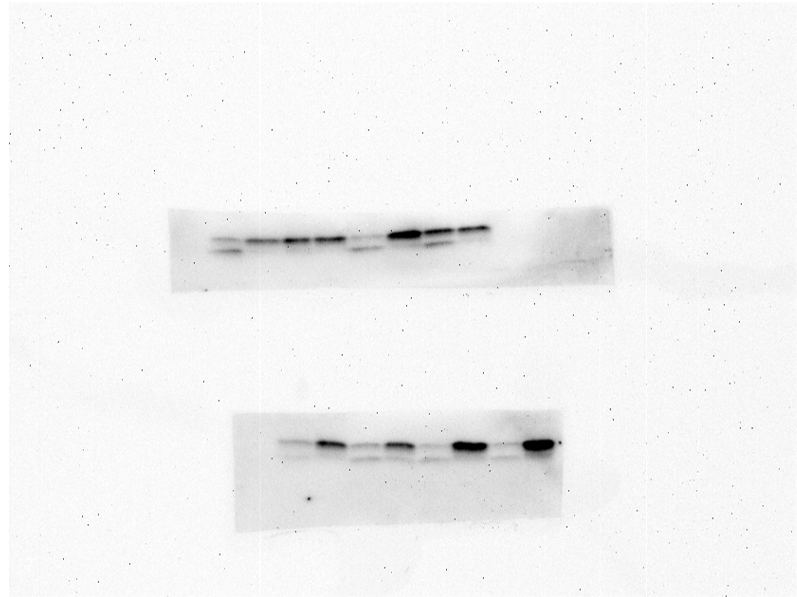

Supplement: Figure 6—source data 1. [file elife-85289-fig6-data1.zip › Figure 6-Source Data 1/Figure 6B LC3.png]

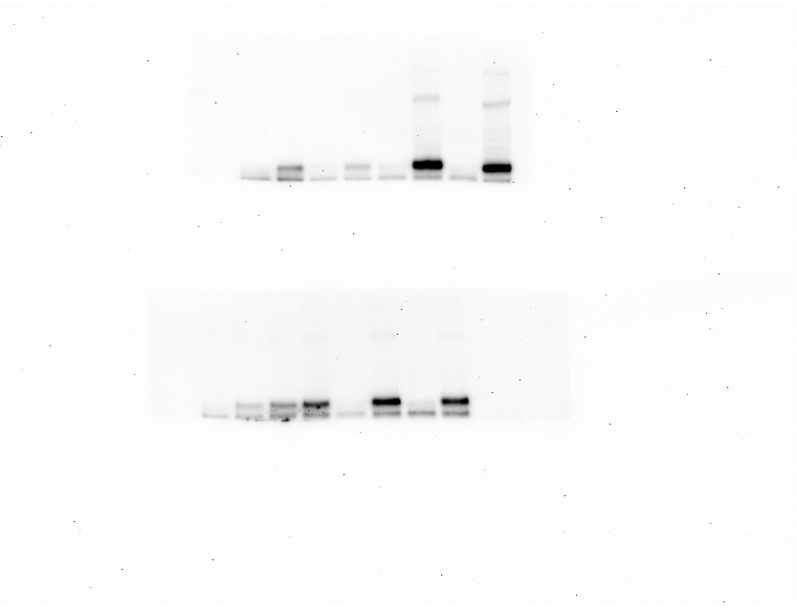

Supplement: Figure 6—source data 1. [file elife-85289-fig6-data1.zip › Figure 6-Source Data 1/Figure 6B p62.png]

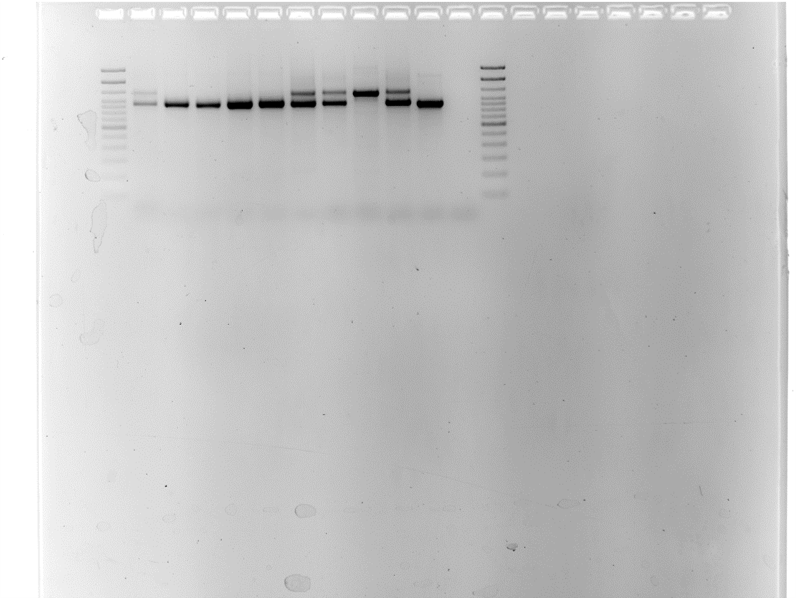

Supplement: Figure 6—figure supplement 1—source data 1. [file elife-85289-fig6-figsupp1-data1.zip › Figure 6-supplemental figure 1 - Source Data 1/Figure 6S1A ATG3cKO LoxP Genotyping.png]

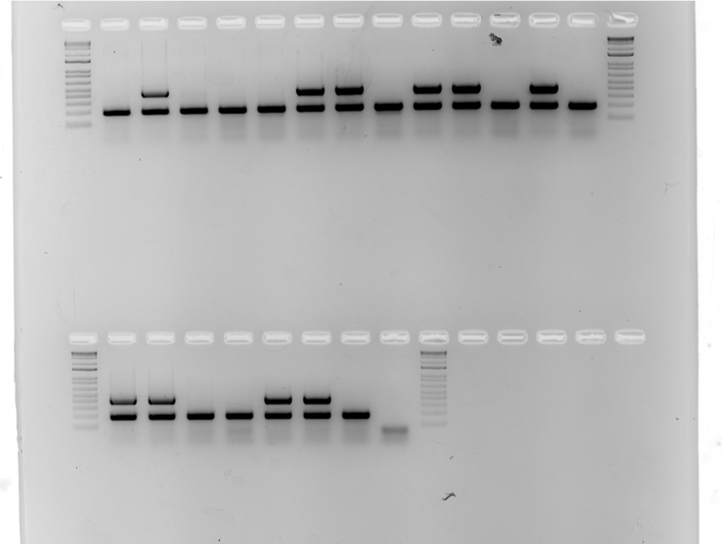

Supplement: Figure 6—figure supplement 1—source data 1. [file elife-85289-fig6-figsupp1-data1.zip › Figure 6-supplemental figure 1 - Source Data 1/Figure 6S1A Cre Genotyping.png]

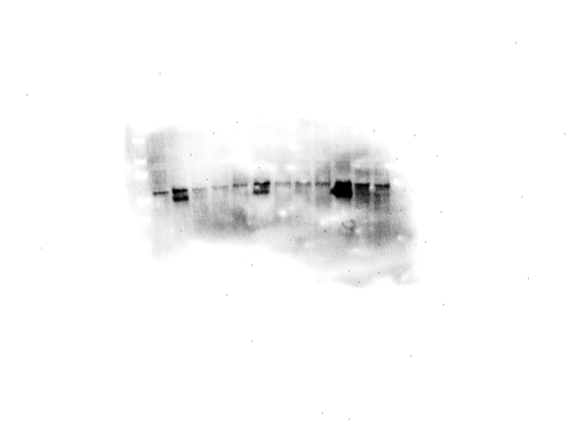

Supplement: Figure 7—source data 1. [file elife-85289-fig7-data1.zip › Figure 7-Source Data 1/Figure 7A 4HNE.png]

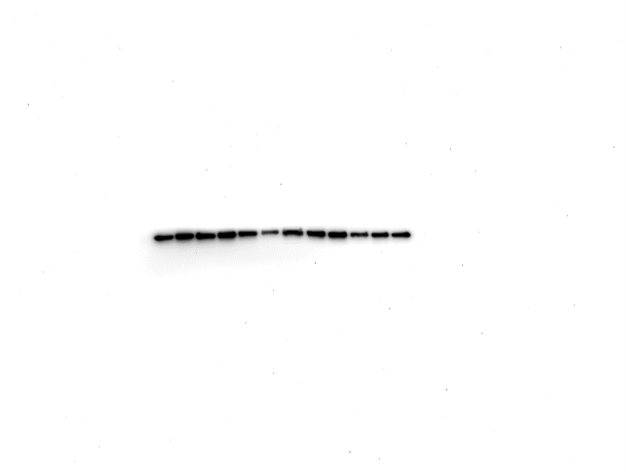

Supplement: Figure 7—source data 1. [file elife-85289-fig7-data1.zip › Figure 7-Source Data 1/Figure 7A Actin.png]

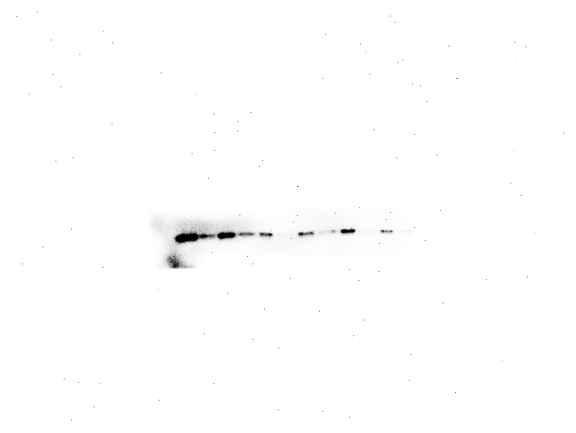

Supplement: Figure 7—source data 1. [file elife-85289-fig7-data1.zip › Figure 7-Source Data 1/Figure 7A GPx4.png]

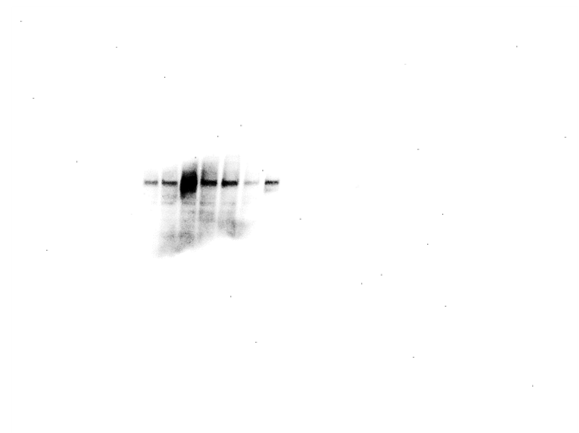

Supplement: Figure 7—source data 1. [file elife-85289-fig7-data1.zip › Figure 7-Source Data 1/Figure 7C 4HNE.png]

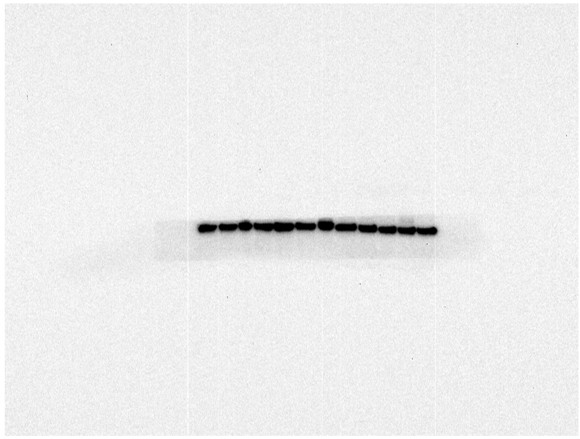

Supplement: Figure 7—source data 1. [file elife-85289-fig7-data1.zip › Figure 7-Source Data 1/Figure 7C Actin.png]

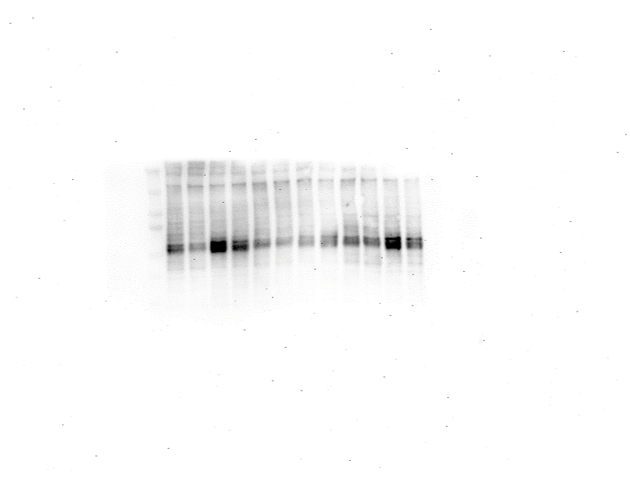

Supplement: Figure 7—source data 1. [file elife-85289-fig7-data1.zip › Figure 7-Source Data 1/Figure 7G 4HNE.png]

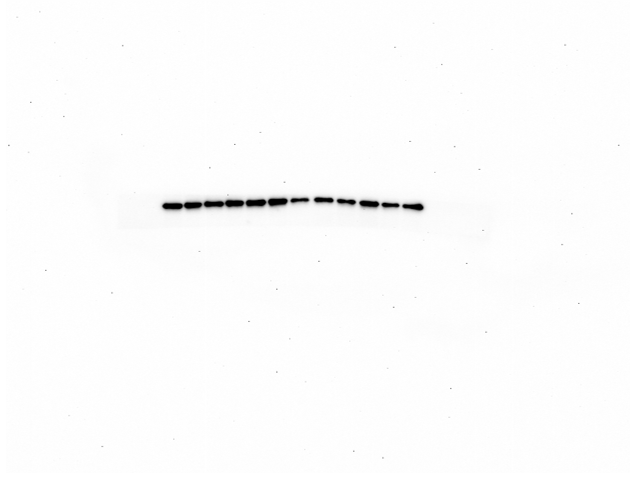

Supplement: Figure 7—source data 1. [file elife-85289-fig7-data1.zip › Figure 7-Source Data 1/Figure 7G Actin.png]

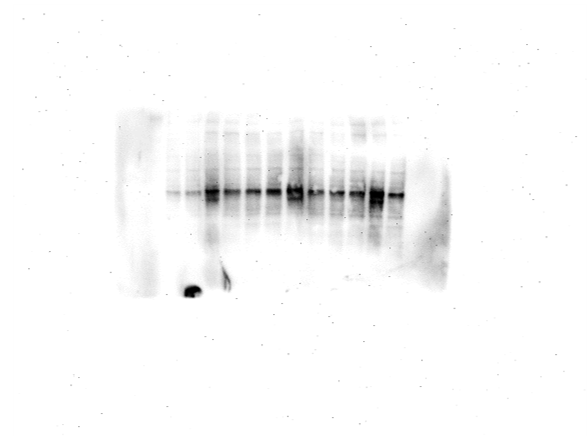

Supplement: Figure 7—figure supplement 1—source data 1. [file elife-85289-fig7-figsupp1-data1.zip › Figure 7-supplemental figure 1 - Source Data 1/Figure 7S1A 4HNE.png]

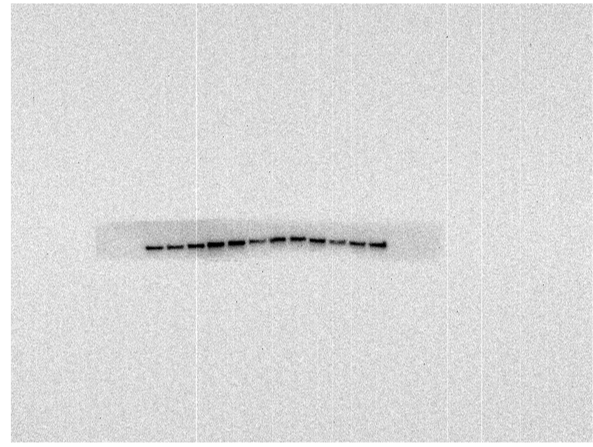

Supplement: Figure 7—figure supplement 1—source data 1. [file elife-85289-fig7-figsupp1-data1.zip › Figure 7-supplemental figure 1 - Source Data 1/Figure 7S1A Actin.png]

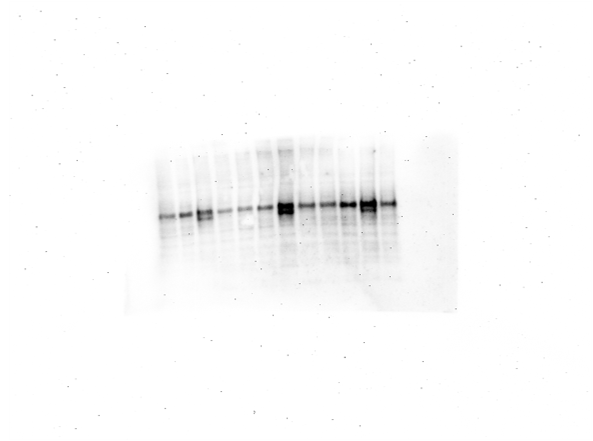

Supplement: Figure 7—figure supplement 1—source data 1. [file elife-85289-fig7-figsupp1-data1.zip › Figure 7-supplemental figure 1 - Source Data 1/Figure 7S1C 4HNE.png]

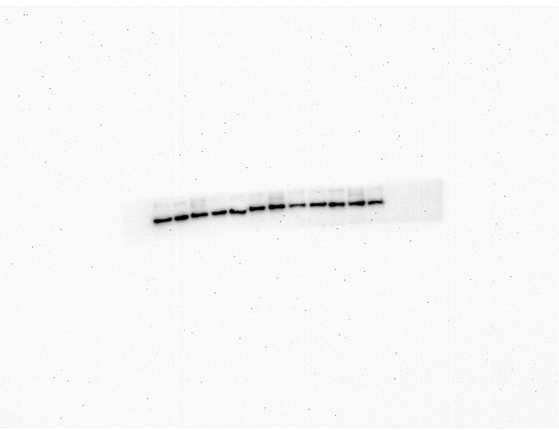

Supplement: Figure 7—figure supplement 1—source data 1. [file elife-85289-fig7-figsupp1-data1.zip › Figure 7-supplemental figure 1 - Source Data 1/Figure 7S1C Actin.png]

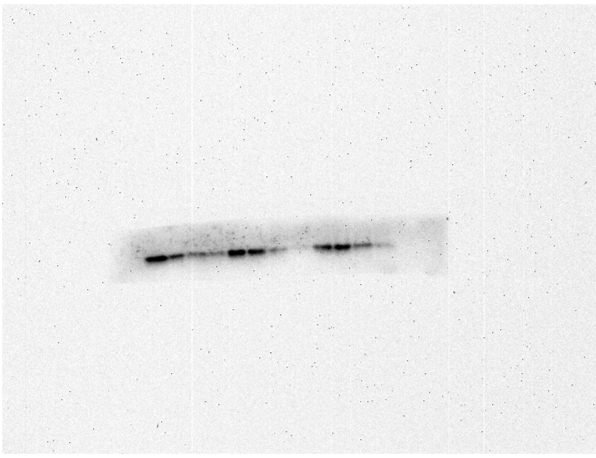

Supplement: Figure 7—figure supplement 1—source data 1. [file elife-85289-fig7-figsupp1-data1.zip › Figure 7-supplemental figure 1 - Source Data 1/Figure 7S1C GPx4.png]

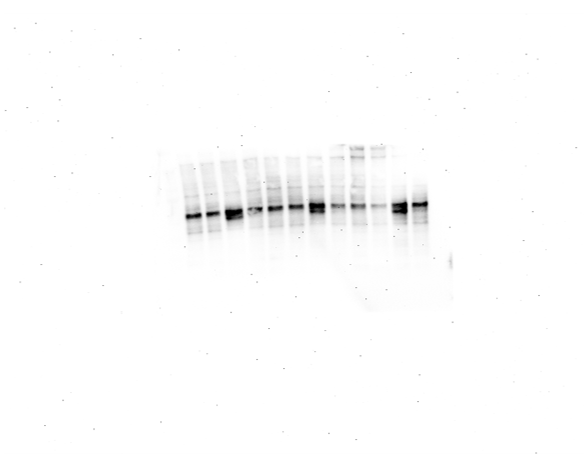

Supplement: Figure 7—figure supplement 1—source data 1. [file elife-85289-fig7-figsupp1-data1.zip › Figure 7-supplemental figure 1 - Source Data 1/Figure 7S1F 4HNE.png]

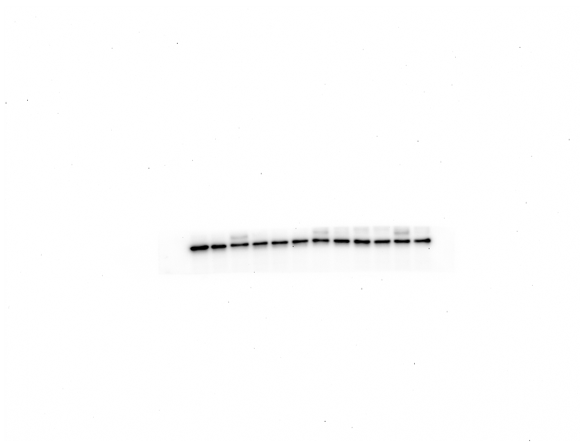

Supplement: Figure 7—figure supplement 1—source data 1. [file elife-85289-fig7-figsupp1-data1.zip › Figure 7-supplemental figure 1 - Source Data 1/Figure 7S1F Actin.png]

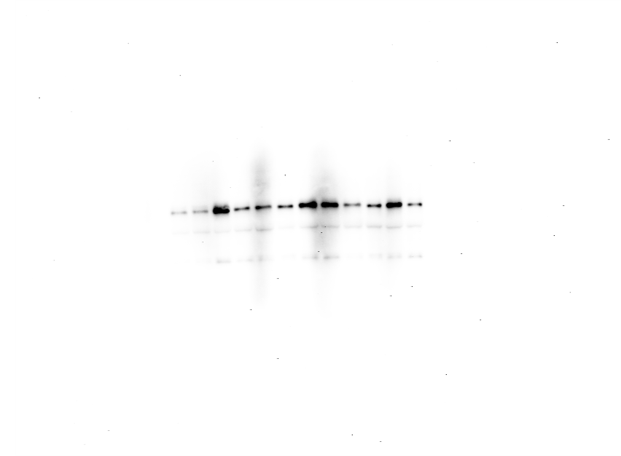

Supplement: Figure 8—source data 1. [file elife-85289-fig8-data1.zip › Figure 8-Source Data 1/Figure 8C 4HNE.png]

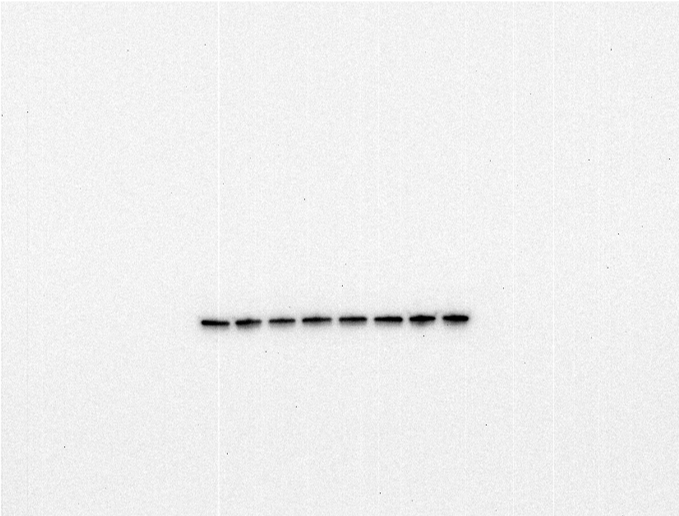

Supplement: Figure 8—source data 1. [file elife-85289-fig8-data1.zip › Figure 8-Source Data 1/Figure 8C GAPDH.png]

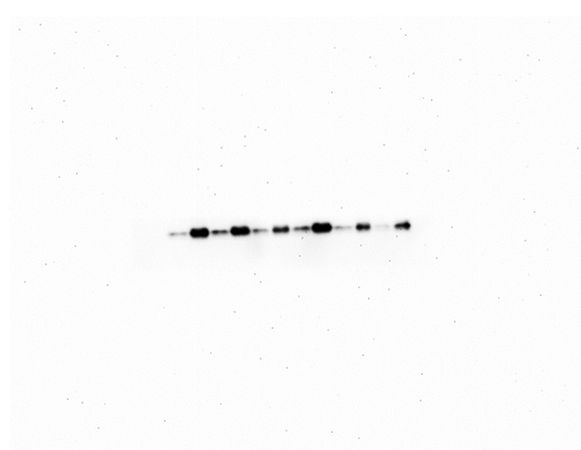

Supplement: Figure 8—source data 1. [file elife-85289-fig8-data1.zip › Figure 8-Source Data 1/Figure 8C GPX4.png]

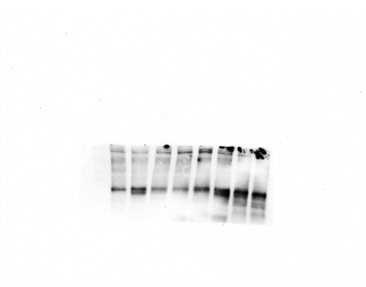

Supplement: Figure 8—source data 1. [file elife-85289-fig8-data1.zip › Figure 8-Source Data 1/Figure 8F 4HNE.png]

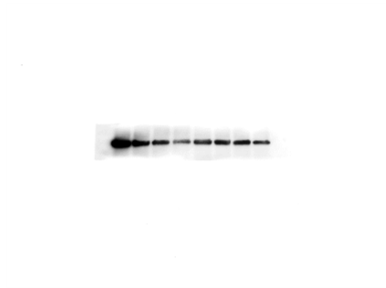

Supplement: Figure 8—source data 1. [file elife-85289-fig8-data1.zip › Figure 8-Source Data 1/Figure 8F Actin.png]

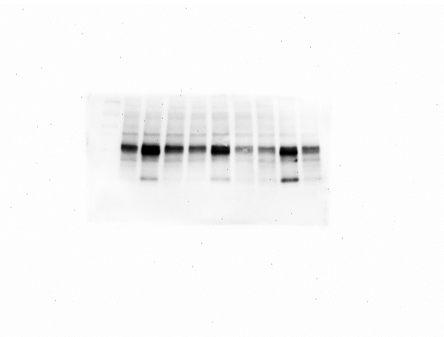

Supplement: Figure 8—figure supplement 2—source data 1. [file elife-85289-fig8-figsupp2-data1.zip › Figure 8-supplemental figure 2 - Source Data 1/Figure 8S2D 4HNE.png]

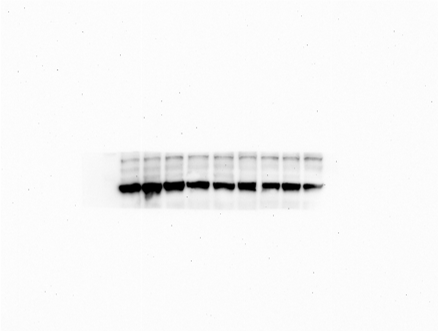

Supplement: Figure 8—figure supplement 2—source data 1. [file elife-85289-fig8-figsupp2-data1.zip › Figure 8-supplemental figure 2 - Source Data 1/Figure 8S2D Actin.png]

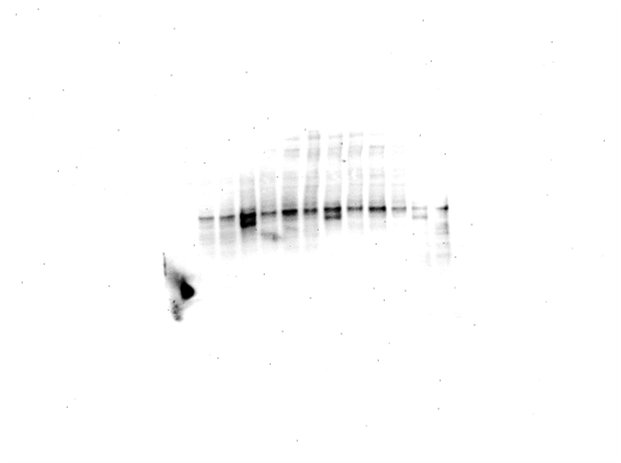

Supplement: Figure 8—figure supplement 2—source data 1. [file elife-85289-fig8-figsupp2-data1.zip › Figure 8-supplemental figure 2 - Source Data 1/Figure 8S2G 4HNE.png]

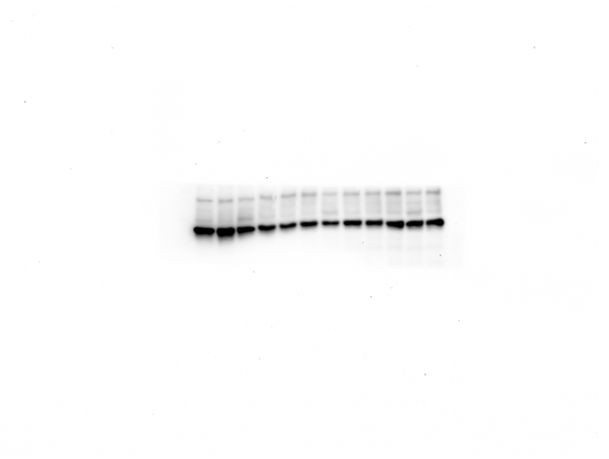

Supplement: Figure 8—figure supplement 2—source data 1. [file elife-85289-fig8-figsupp2-data1.zip › Figure 8-supplemental figure 2 - Source Data 1/Figure 8S2G Actin.png]

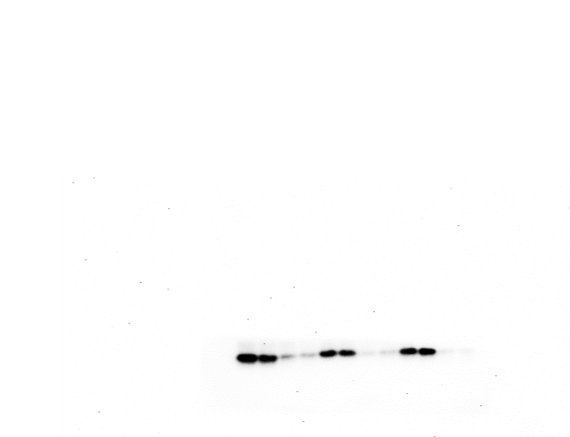

Supplement: Figure 8—figure supplement 2—source data 1. [file elife-85289-fig8-figsupp2-data1.zip › Figure 8-supplemental figure 2 - Source Data 1/Figure 8S2G GPx4.png]

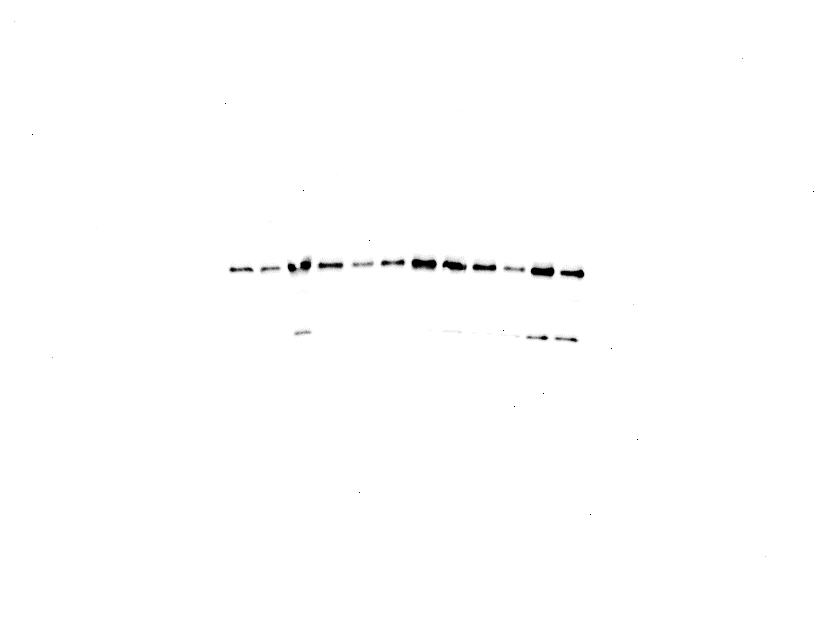

Supplement: Figure 8—figure supplement 3—source data 1. [file elife-85289-fig8-figsupp3-data1.zip › Figure 8-supplemental figure 3 - Source Data 1/Figure S11E 4-HNE.png]

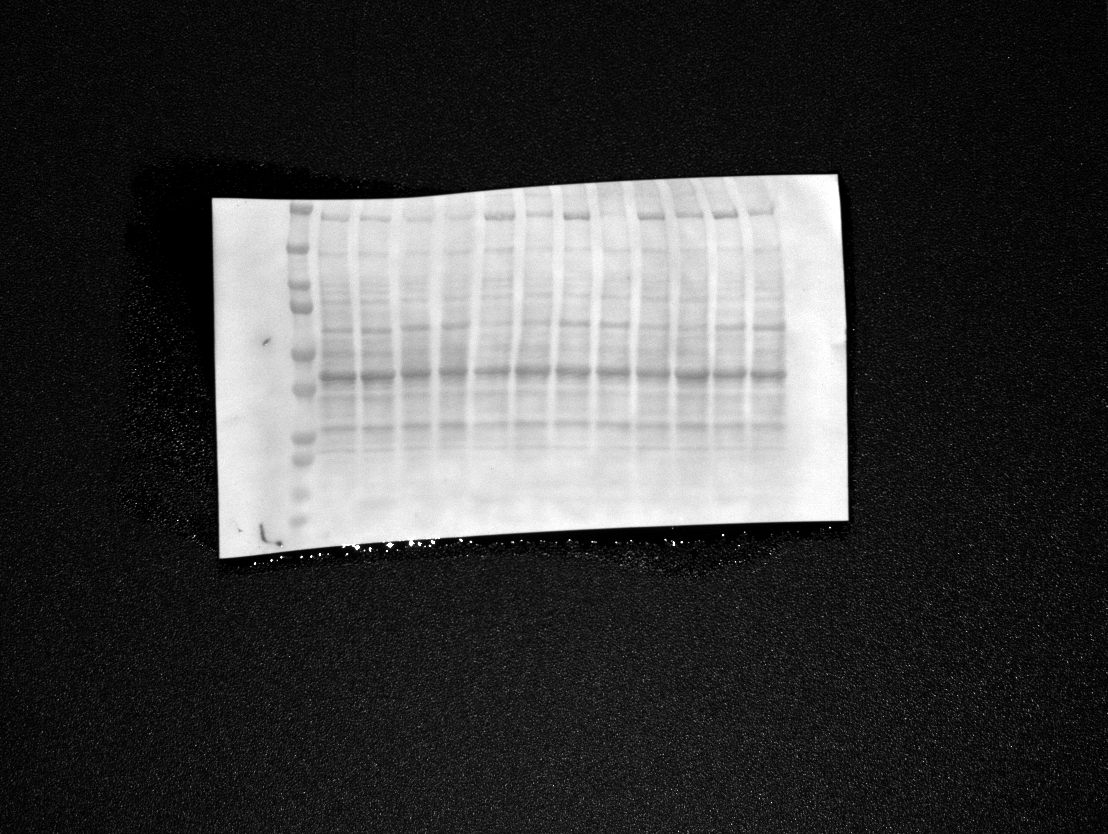

Supplement: Figure 8—figure supplement 3—source data 1. [file elife-85289-fig8-figsupp3-data1.zip › Figure 8-supplemental figure 3 - Source Data 1/Figure S11E Ponceau S.png]

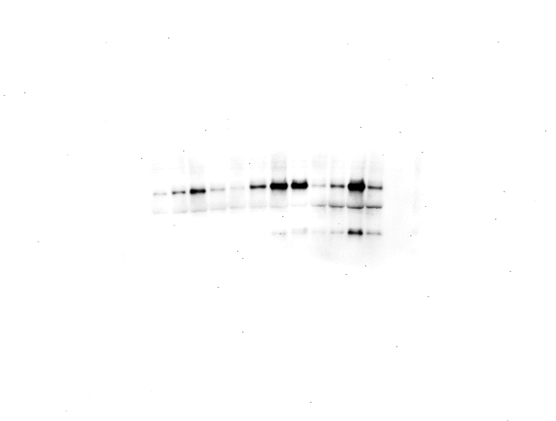

Supplement: Figure 9—source data 1. [file elife-85289-fig9-data1.zip › Figure 9-Source Data 1/Figure 9B 4HNE.png]

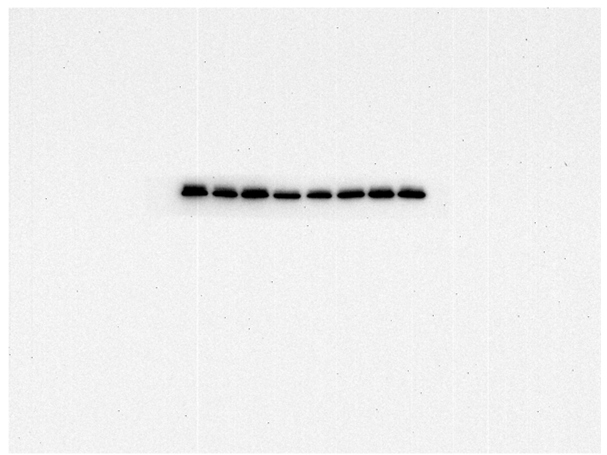

Supplement: Figure 9—source data 1. [file elife-85289-fig9-data1.zip › Figure 9-Source Data 1/Figure 9B GAPDH.png]

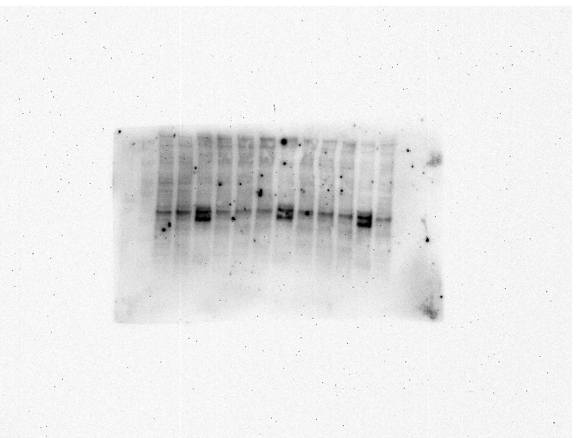

Supplement: Figure 9—figure supplement 1—source data 1. [file elife-85289-fig9-figsupp1-data1.zip › Figure 9-supplemental figure 1 - Source Data 1/Figure 9S1A 4HNE.png]

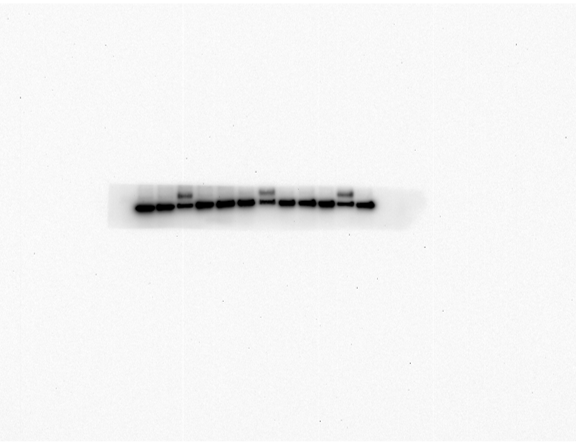

Supplement: Figure 9—figure supplement 1—source data 1. [file elife-85289-fig9-figsupp1-data1.zip › Figure 9-supplemental figure 1 - Source Data 1/Figure 9S1A Actin.png]

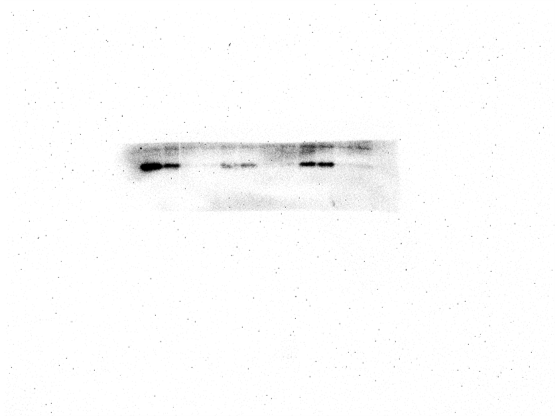

Supplement: Figure 9—figure supplement 1—source data 1. [file elife-85289-fig9-figsupp1-data1.zip › Figure 9-supplemental figure 1 - Source Data 1/Figure 9S1A GPx4.png]

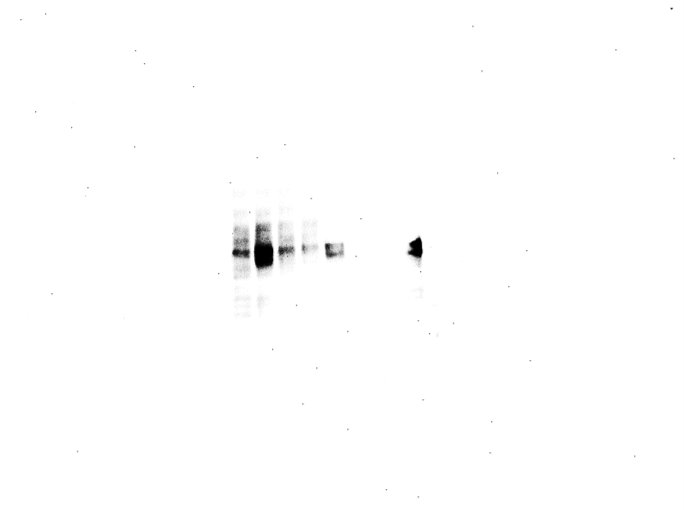

Supplement: Figure 9—figure supplement 1—source data 1. [file elife-85289-fig9-figsupp1-data1.zip › Figure 9-supplemental figure 1 - Source Data 1/Figure 9S1D 4HNE.png]

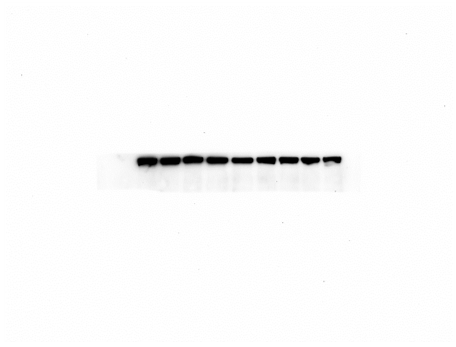

Supplement: Figure 9—figure supplement 1—source data 1. [file elife-85289-fig9-figsupp1-data1.zip › Figure 9-supplemental figure 1 - Source Data 1/Figure 9S1D Actin.png]

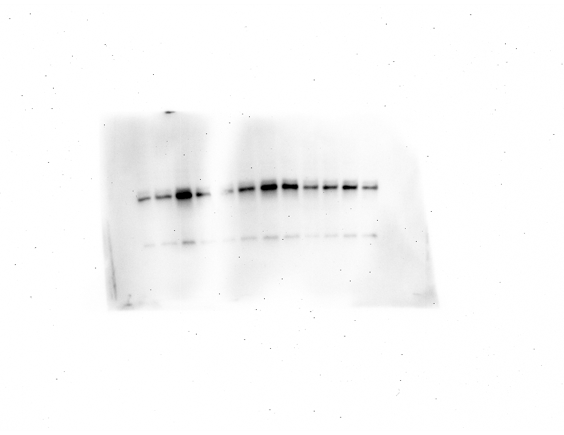

Supplement: Figure 9—figure supplement 2—source data 1. [file elife-85289-fig9-figsupp2-data1.zip › Figure 9-supplemental figure 2 - Source Data 1/Figure 9S2F 4HNE.png]

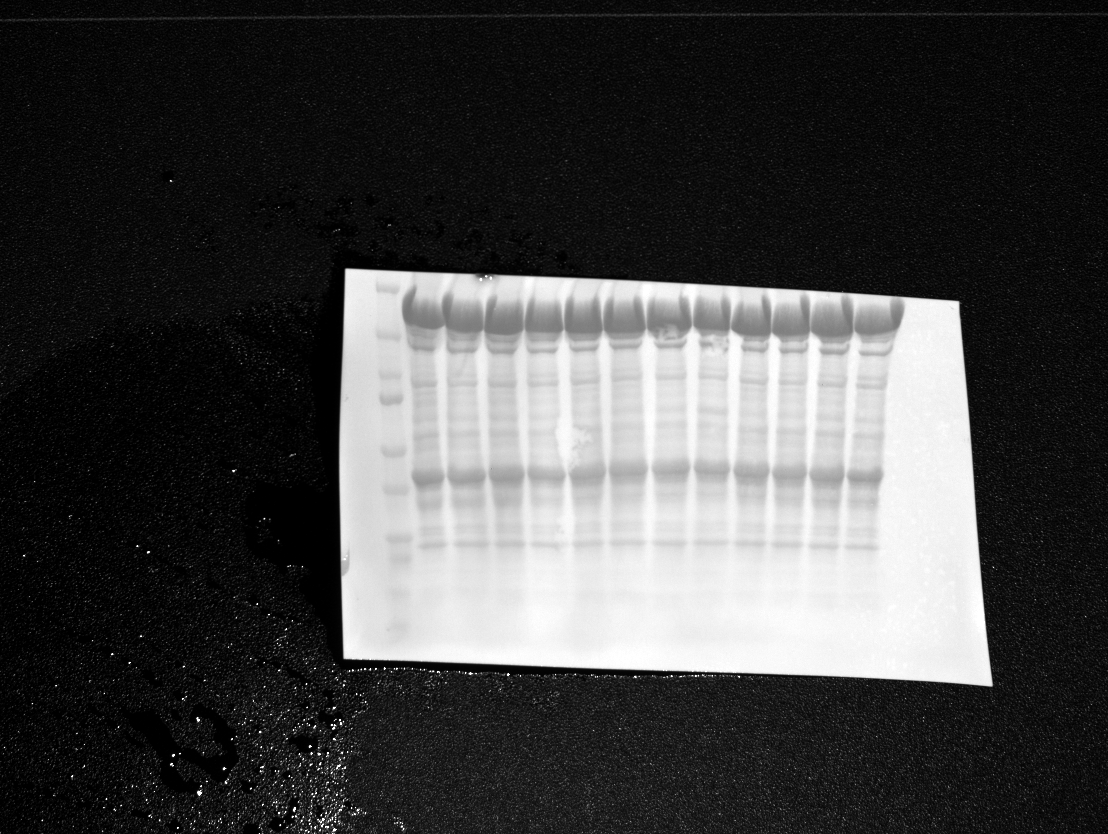

Supplement: Figure 9—figure supplement 2—source data 1. [file elife-85289-fig9-figsupp2-data1.zip › Figure 9-supplemental figure 2 - Source Data 1/Figure 9S2F Ponceau S..png]
