# Supplementary material for: Lipid hydroperoxides promote sarcopenia through carbonyl stress
Source: eLife. 2023 Mar 23;12:e85289. doi: 10.7554/eLife.85289 (PMC10076018; doi:10.7554/eLife.85289)

Figure 1E

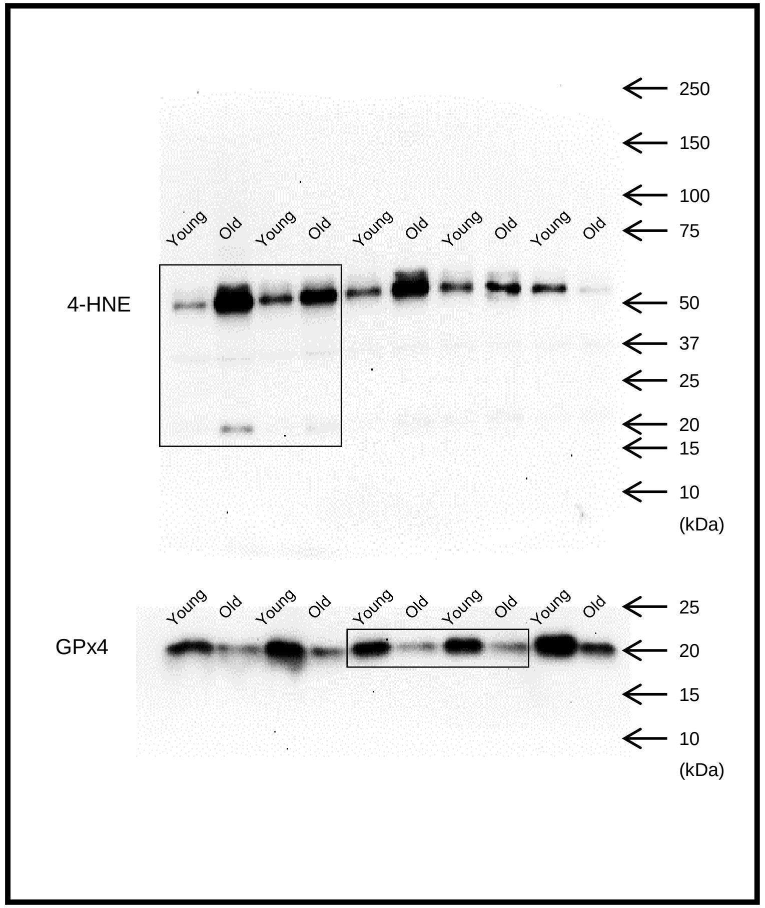

4-HNE
Young
Old
Young
Old
Young
Old
Young
Old
Young
Old
250
150
100
75
50
37
25
20
15
10
(kDa)
GPx4
Young
Old
Young
Old
Young
Old
Young
Old
Young
Old
25
20
15
10
(kDa)

Figure 1-supplemental figure 1F

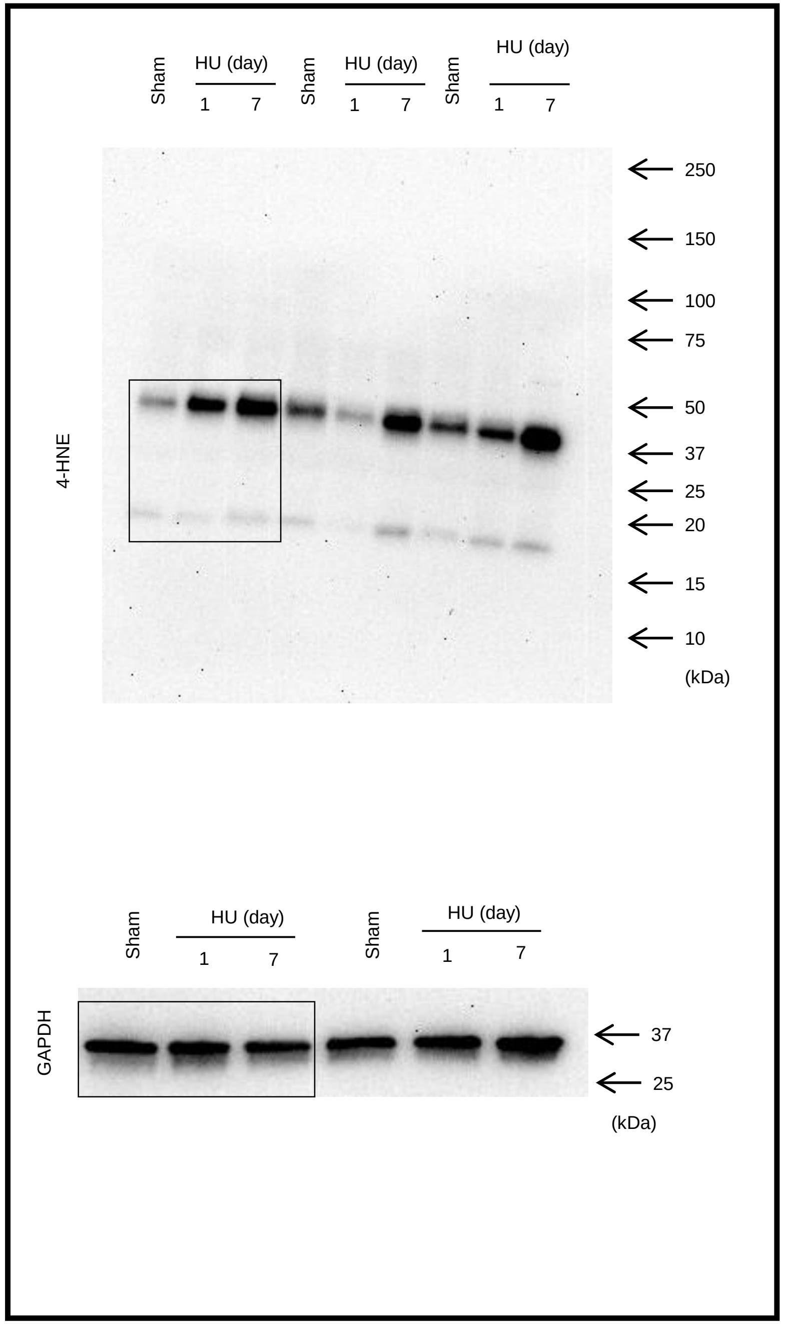

Figure 2E

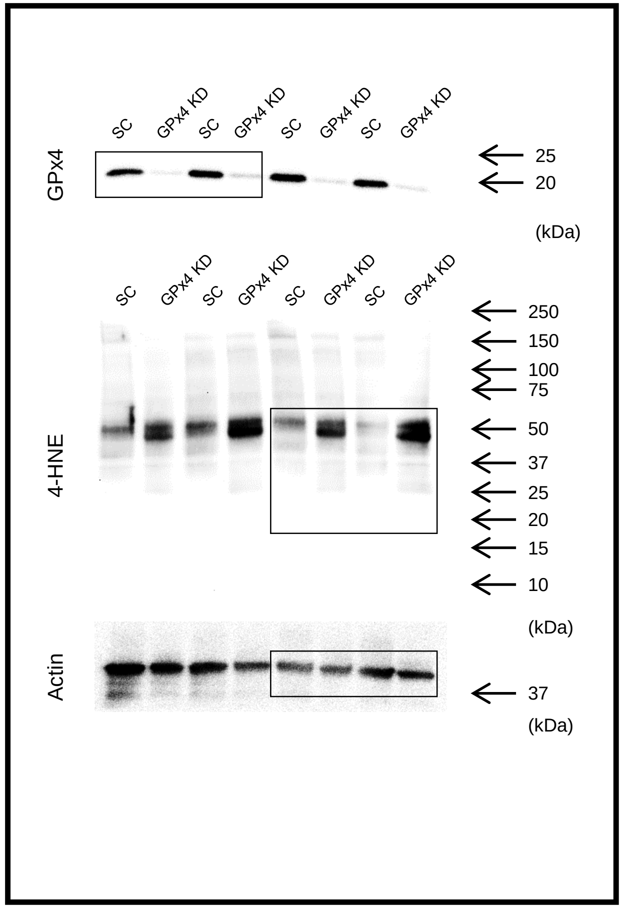
SC
GPx4 KD
SC
GPx4 KD
SC
GPx4 KD
SC
GPx4 KD
GPx4
25
20
(kDa)
4-HNE
250
150
100
75
50
37
25
20
15
10
(kDa)
Actin
37
(kDa)

Figure 2-supplemental figure 1B

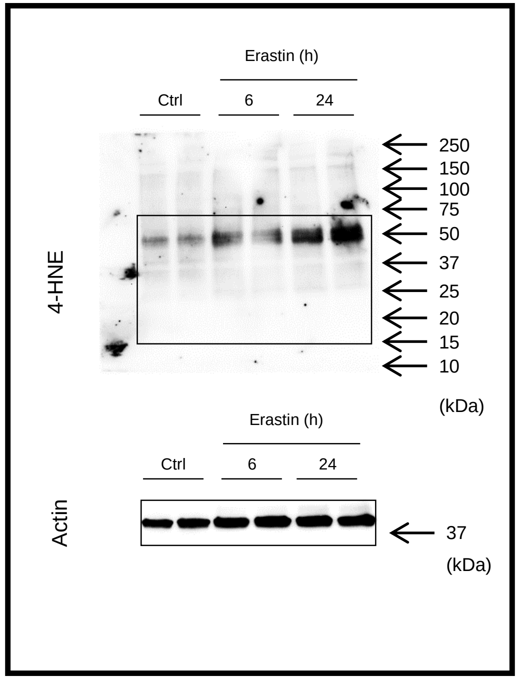

Figure 2-supplemental figure 1G

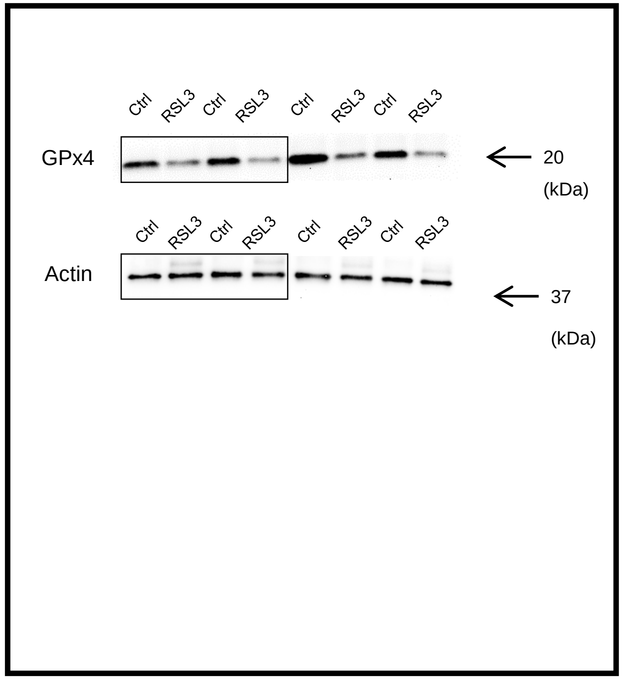

Figure 3B

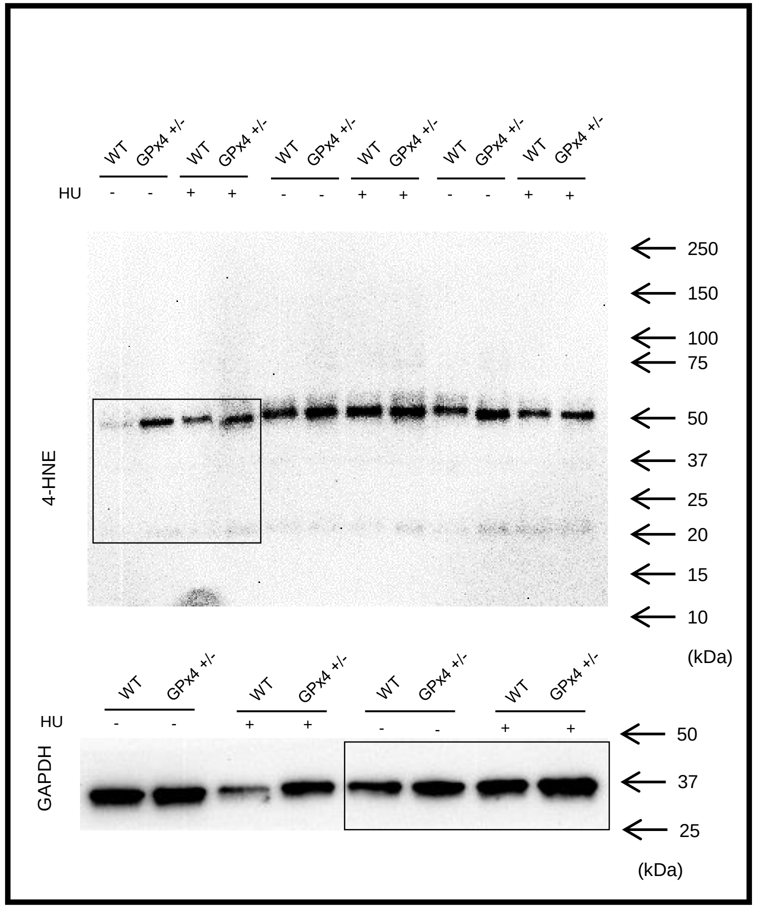

Figure 3G

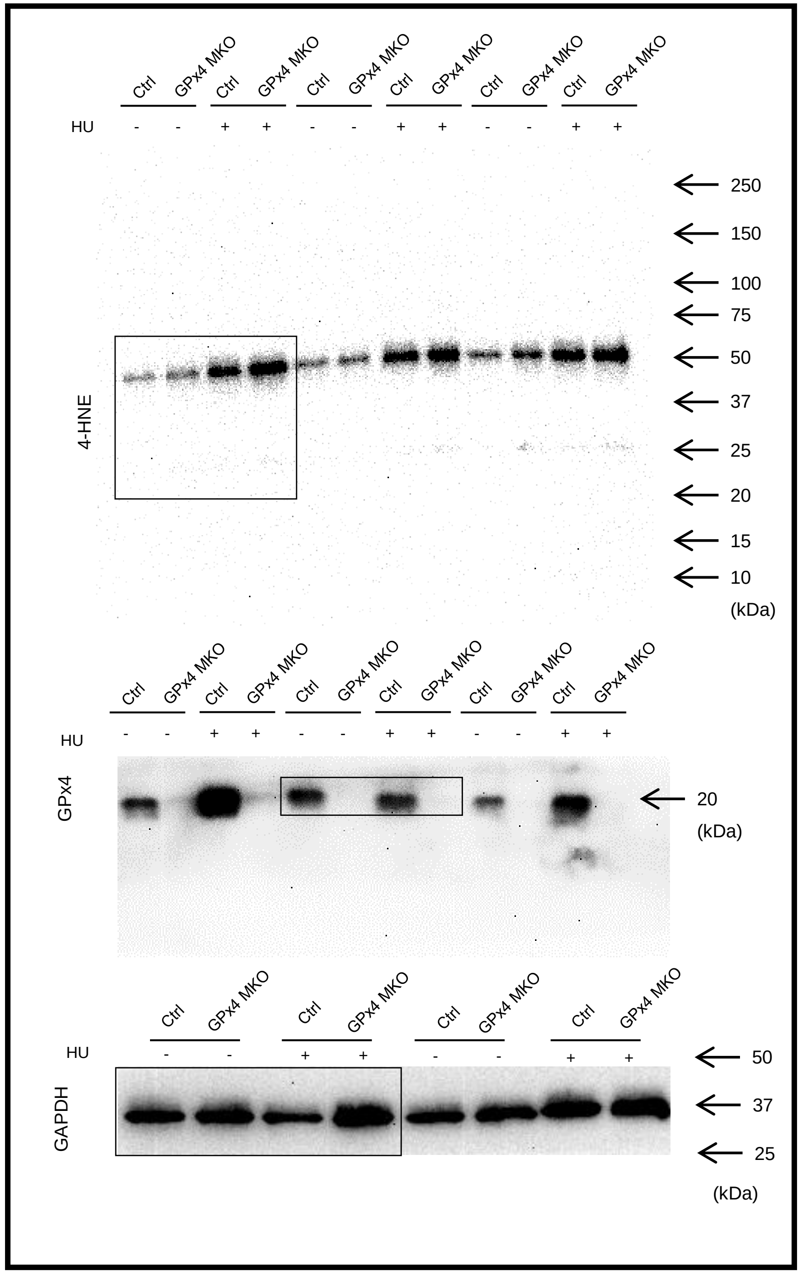

Figure 3-supplemental figure 2A

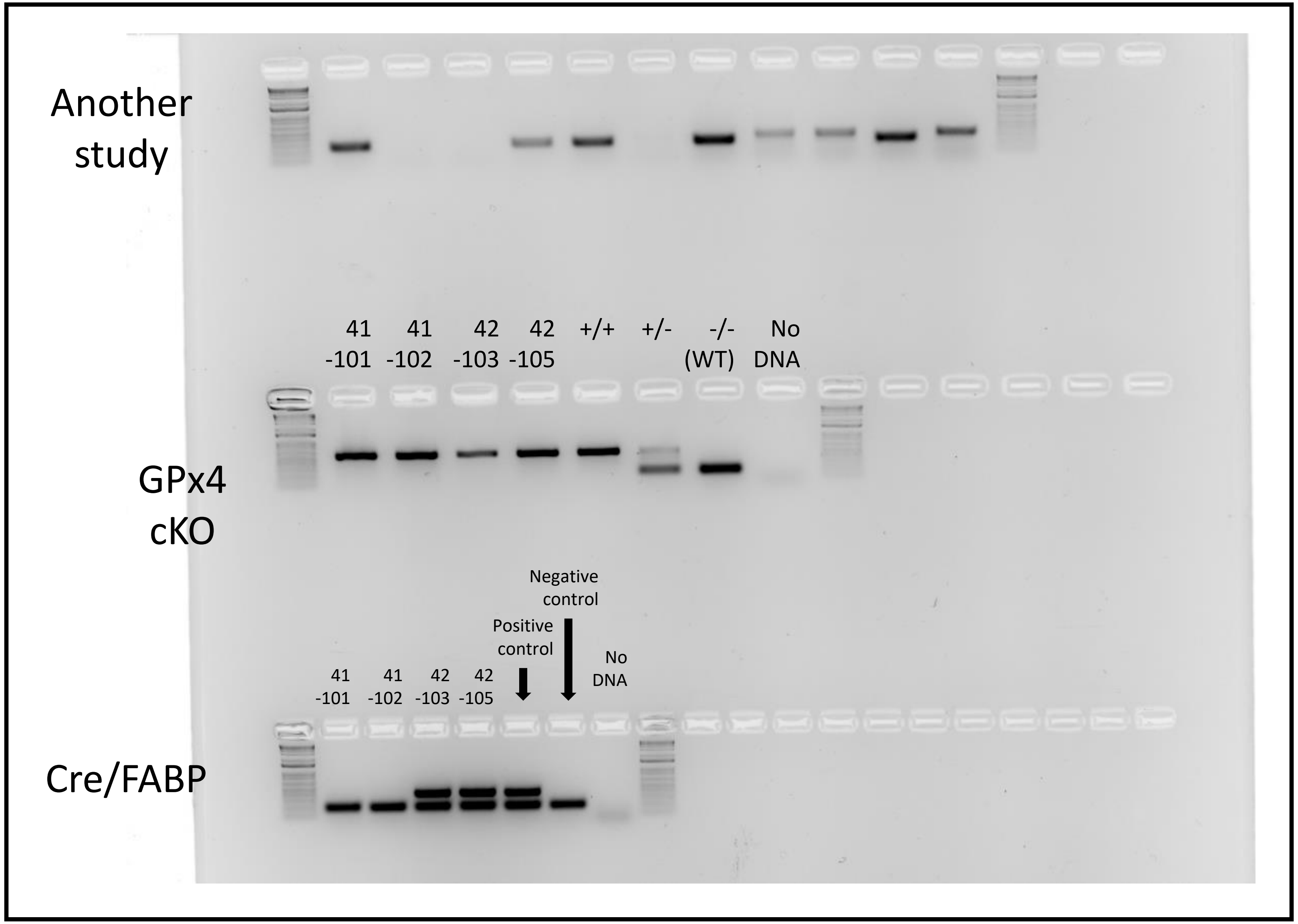

Figure 3-supplemental figure 2A

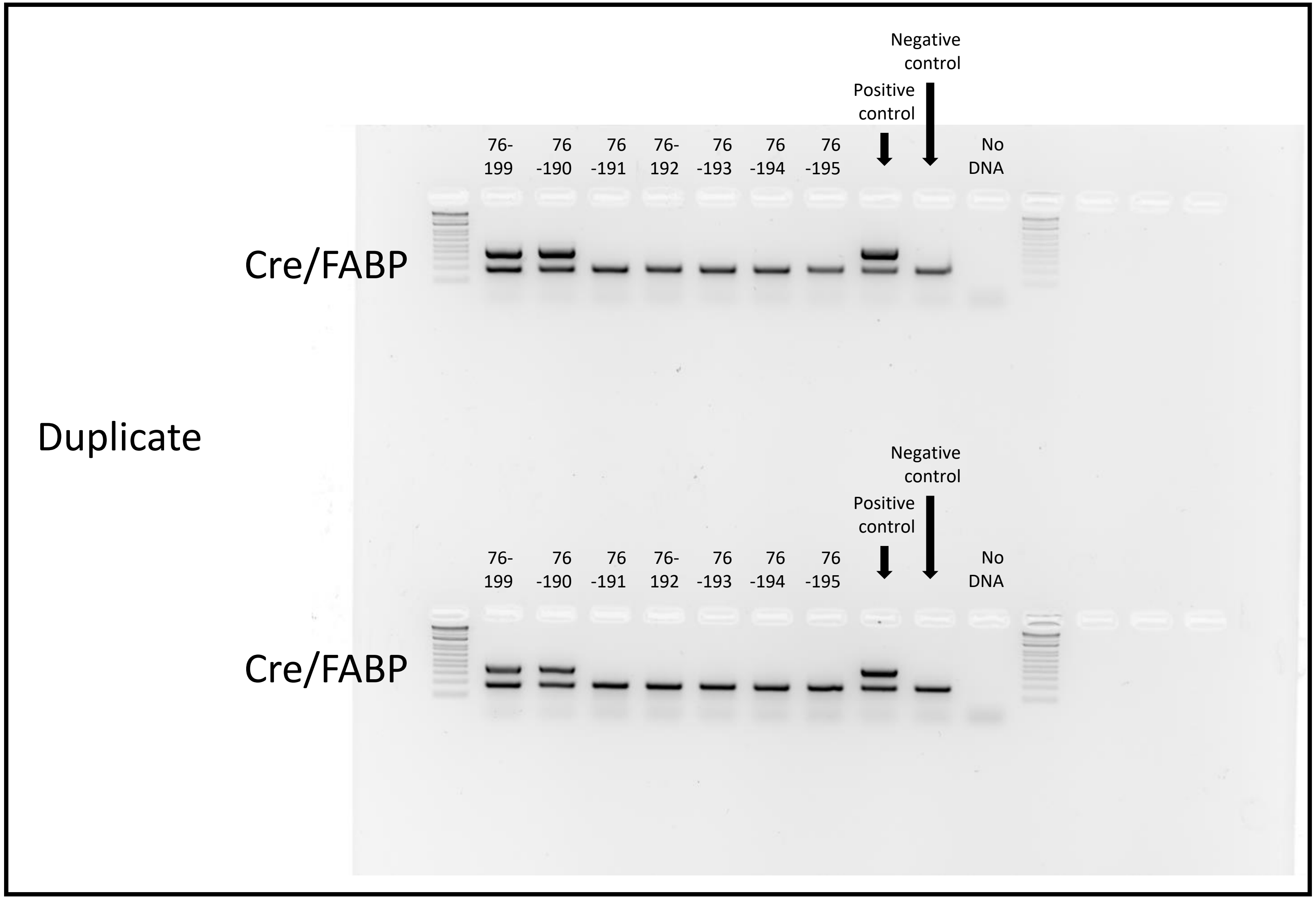

Figure 4B

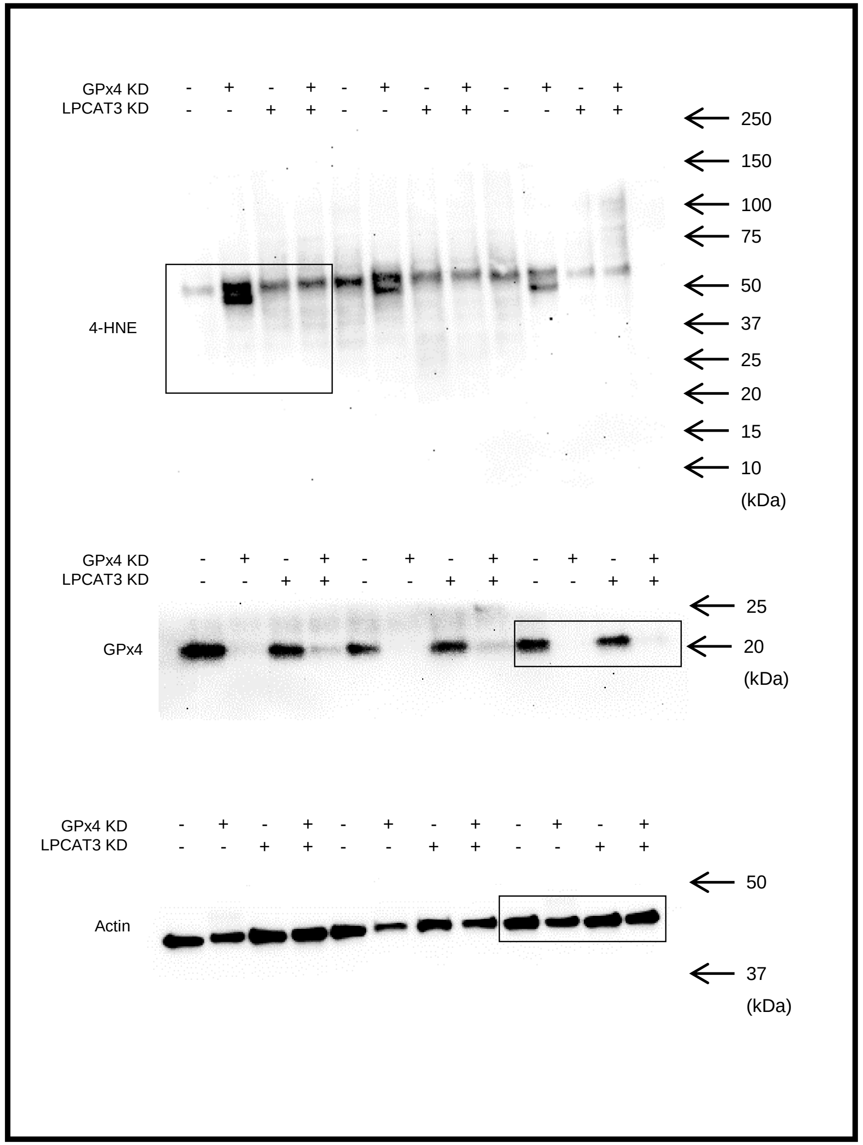

Figure 4F

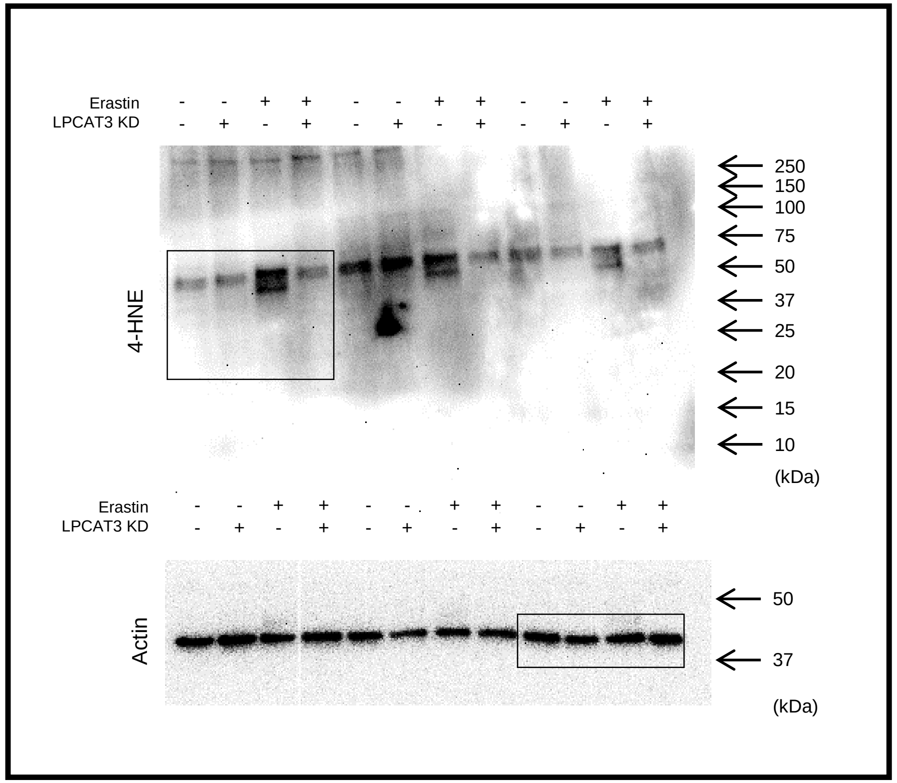

Figure 5c

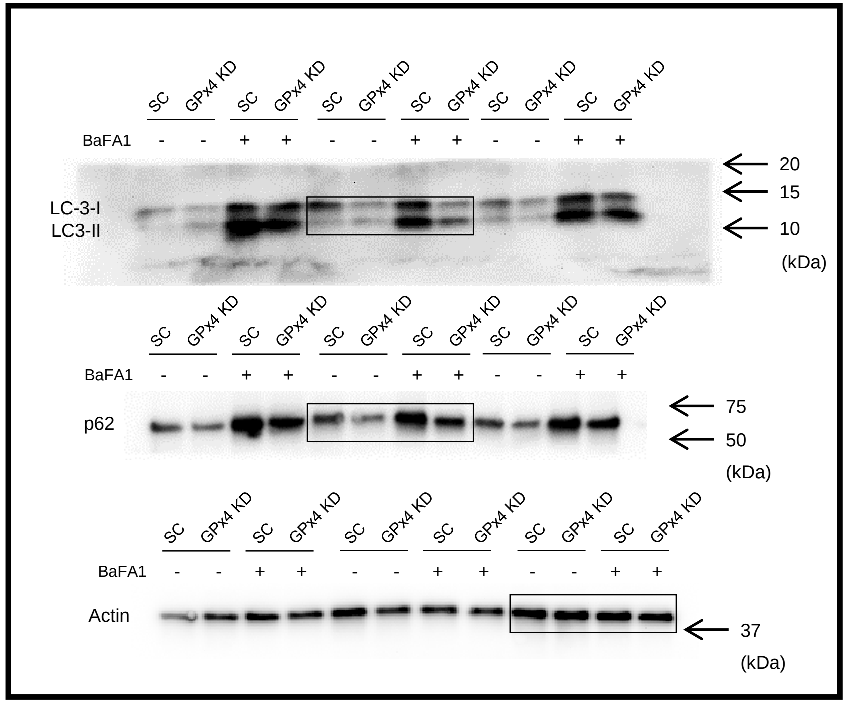
SC
GPx4 KD
BaFA1
- - + + - - + + - - + +
LC-3-I
LC3-II
20
15
10
(kDa)
p62
75
50
(kDa)
Actin
37
(kDa)

Figure 5-supplemental figure 1D

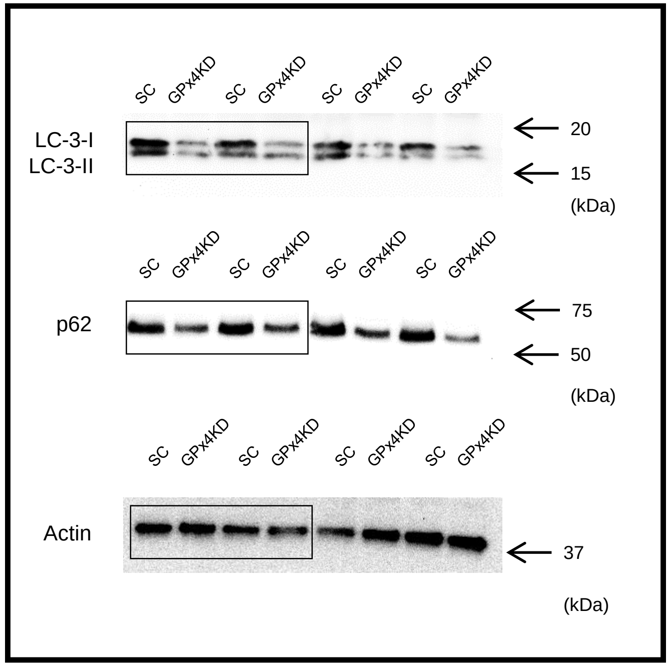

Figure 5-supplemental figure 1K

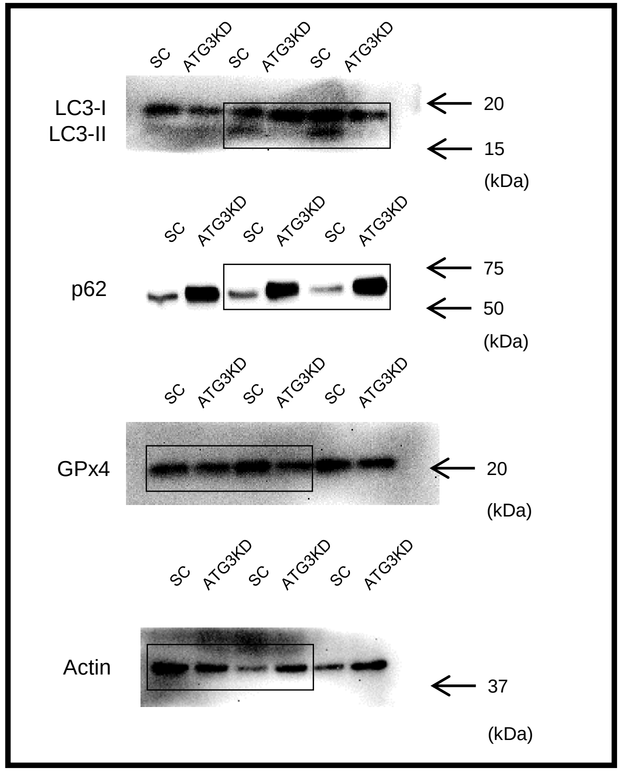

Figure 6B

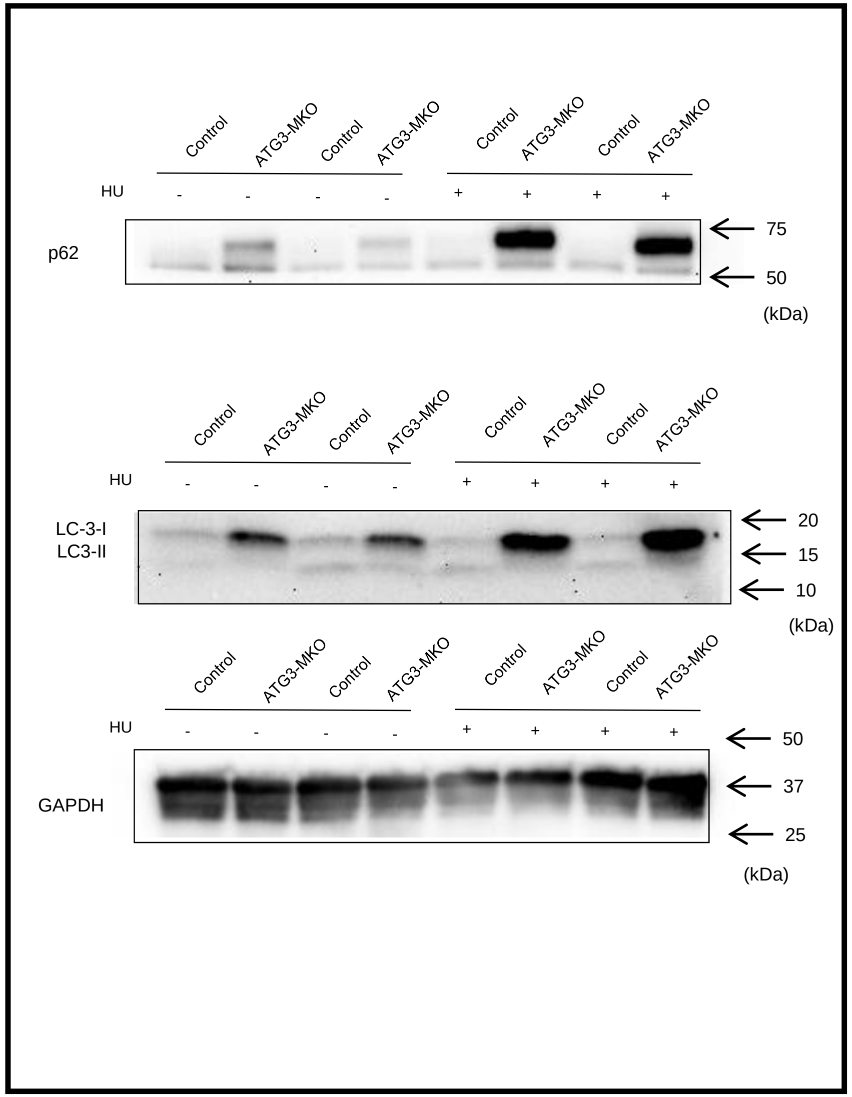

Figure 6B Continued

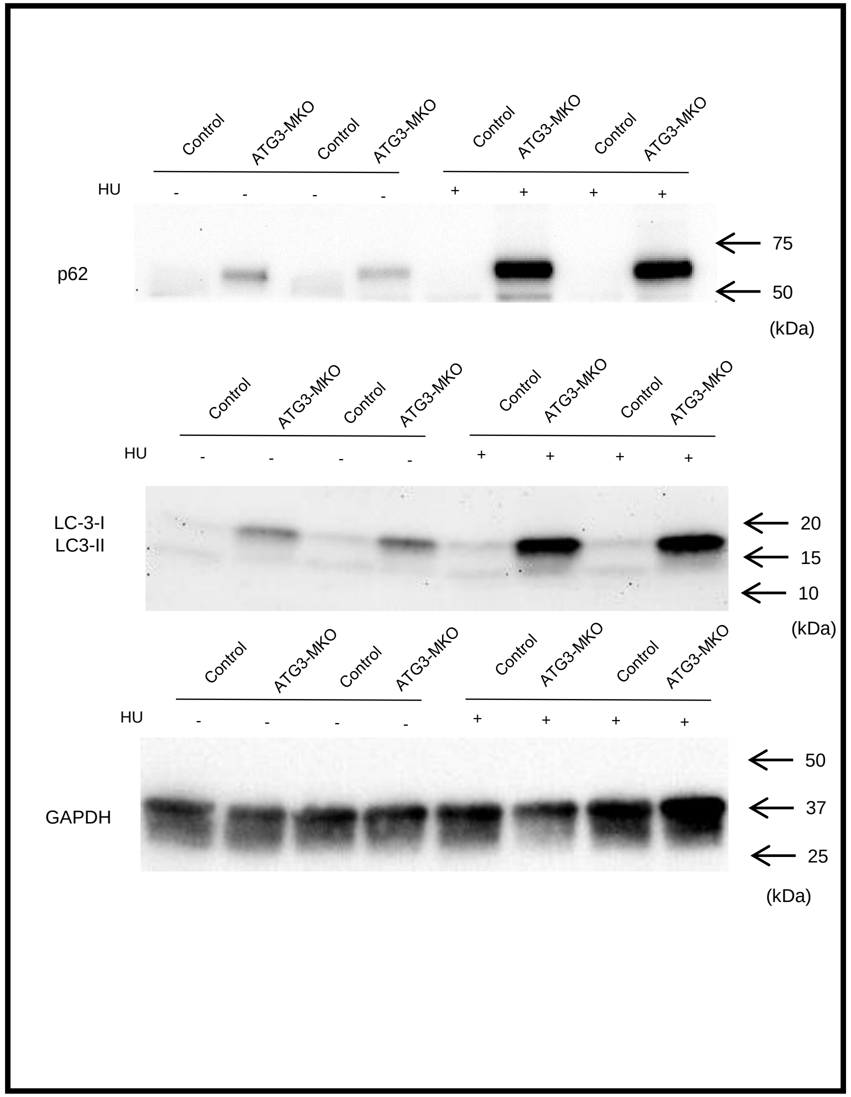

Figure 6B Continued

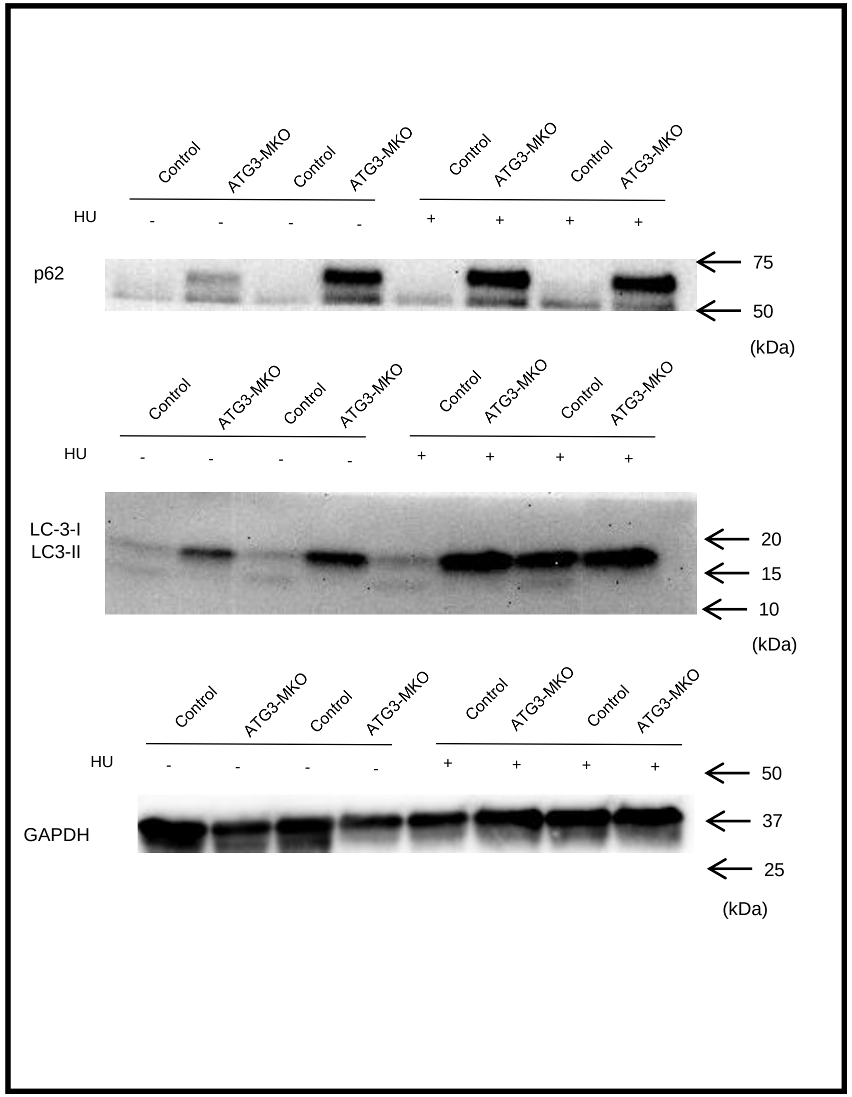

Figure 6B Continued

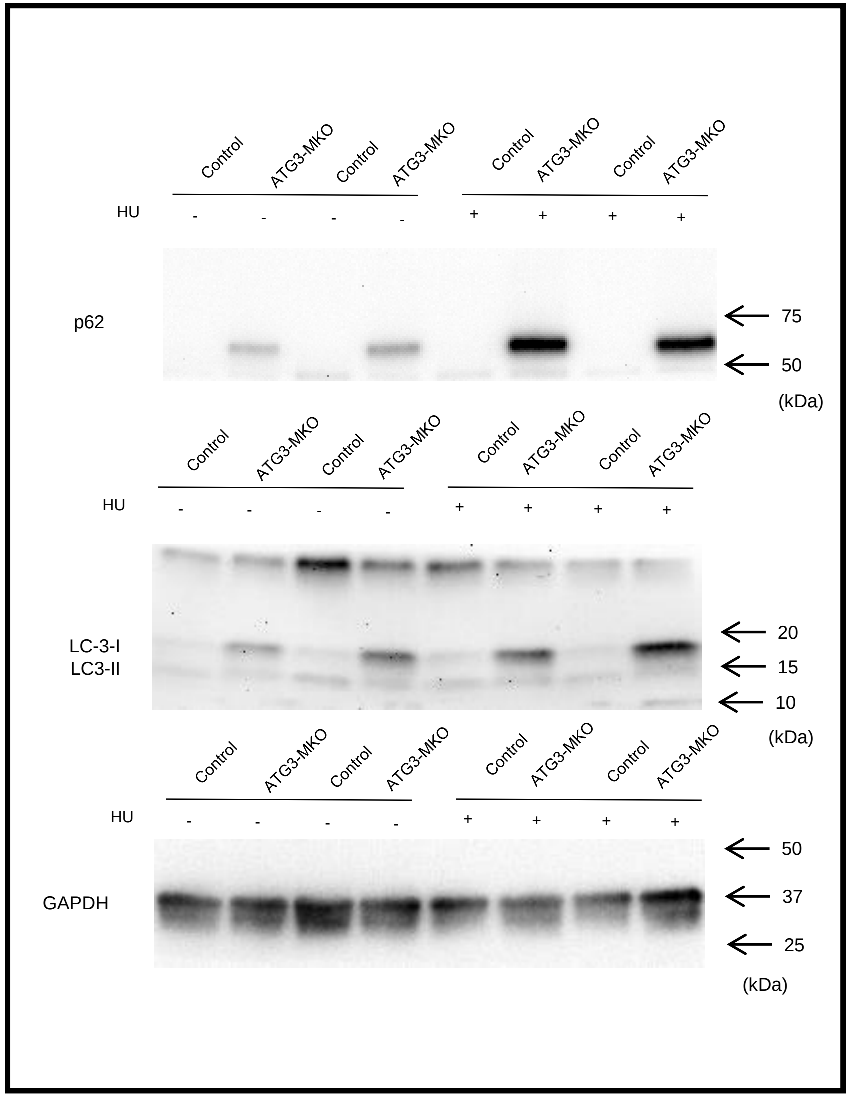

Figure 6-supplemental figure 1A

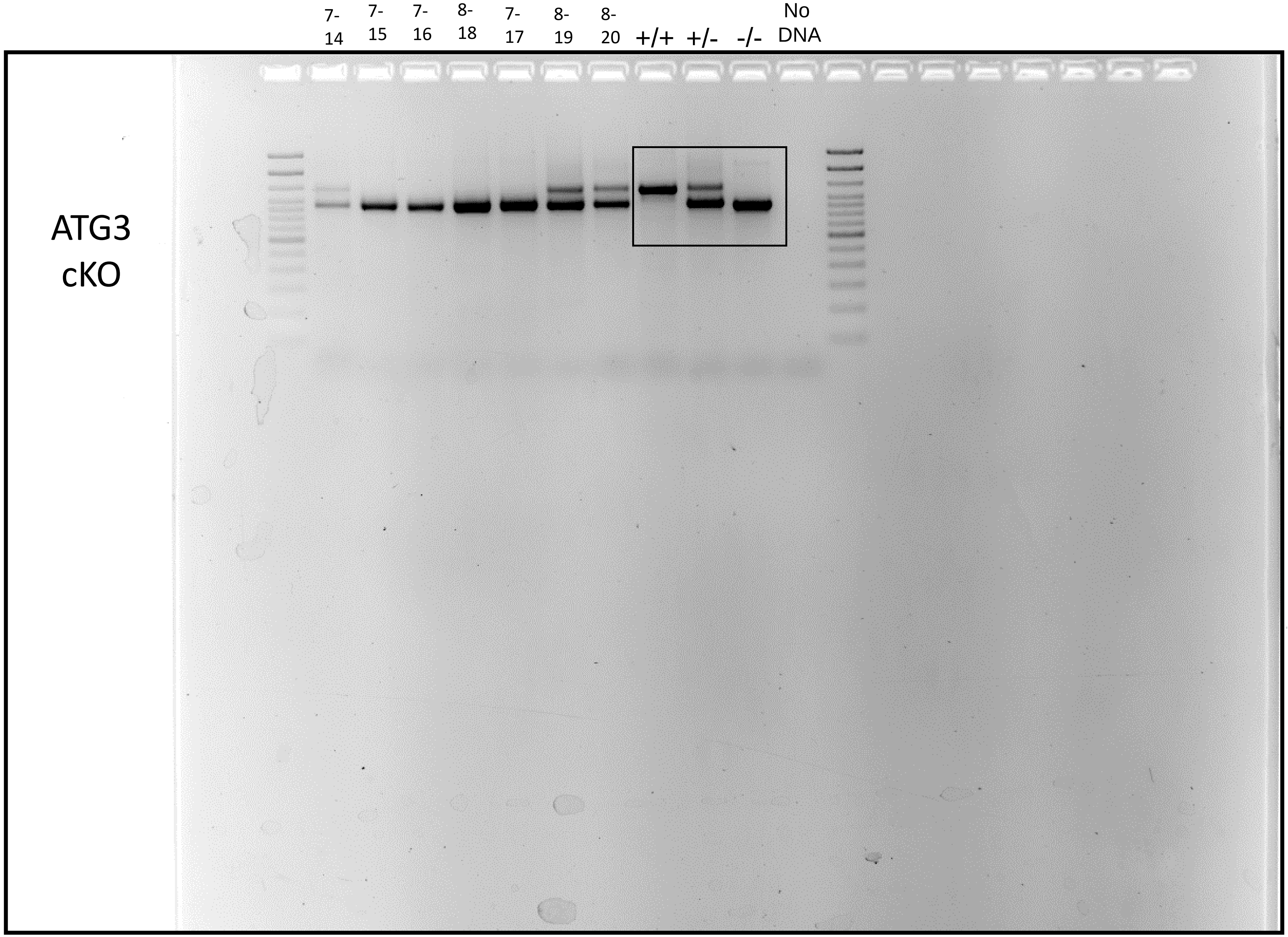

Figure 6-supplemental figure 1A Continued

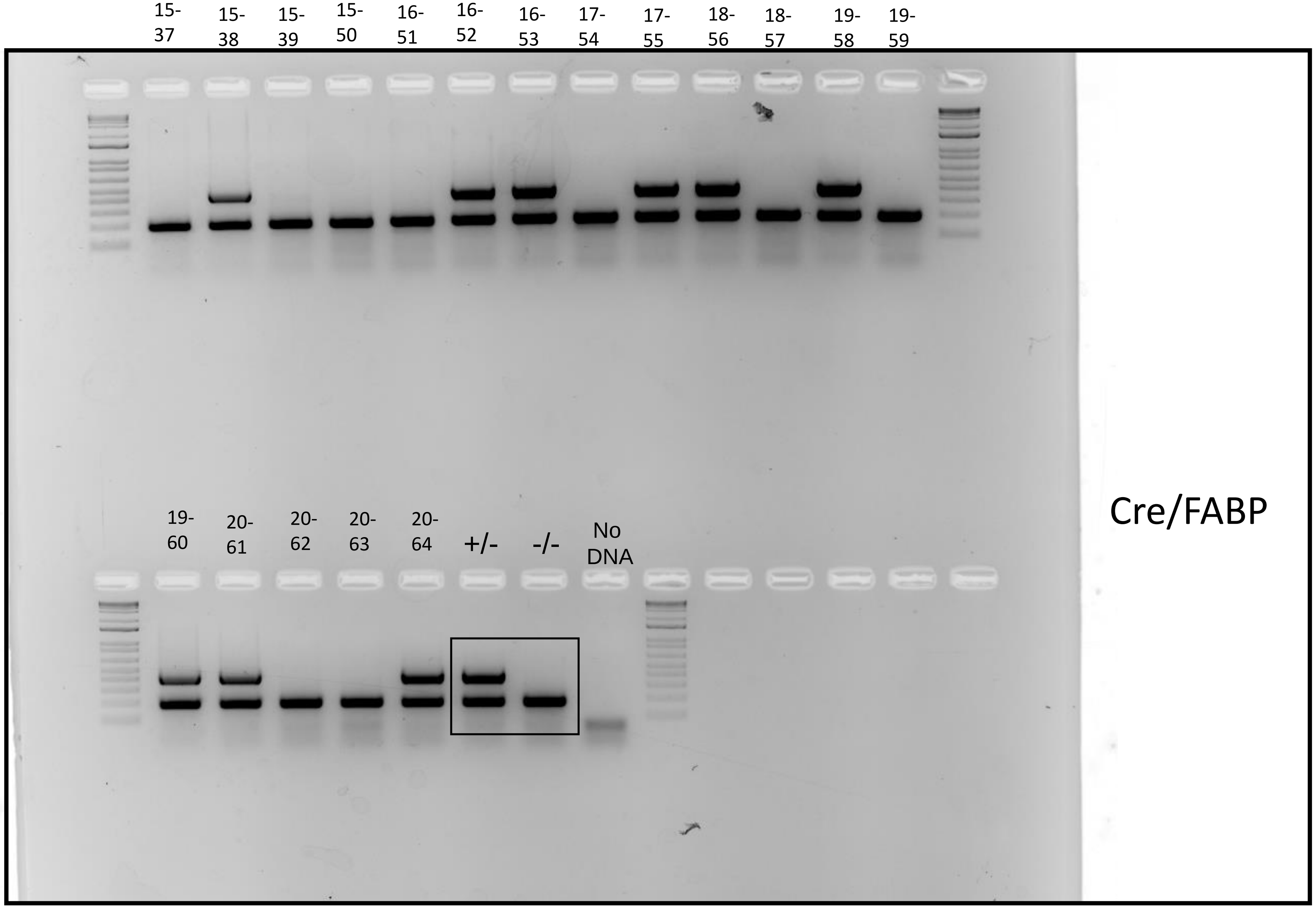

Figure 7A

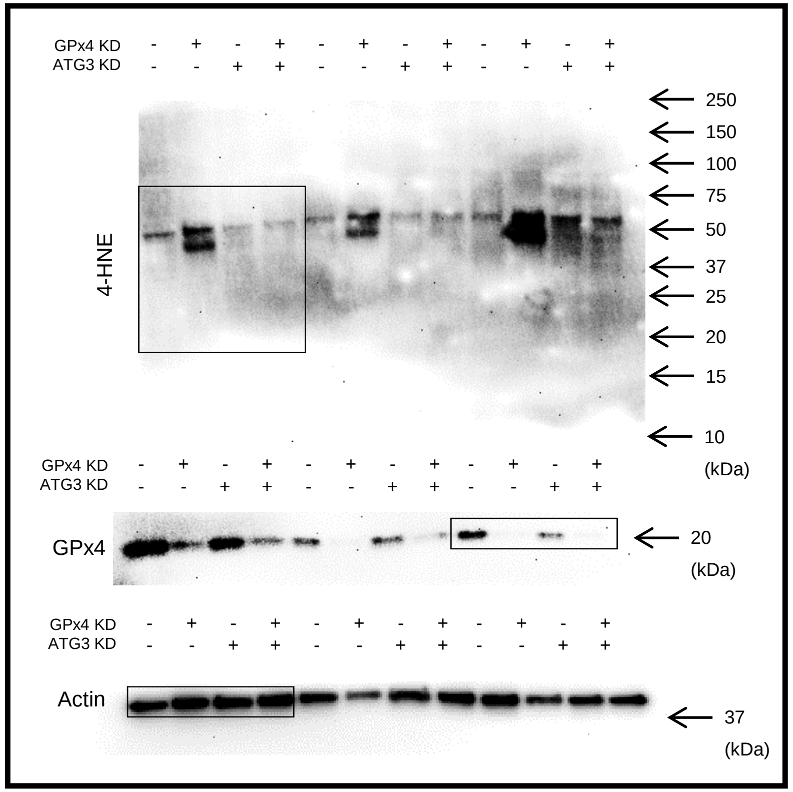

Figure 7C

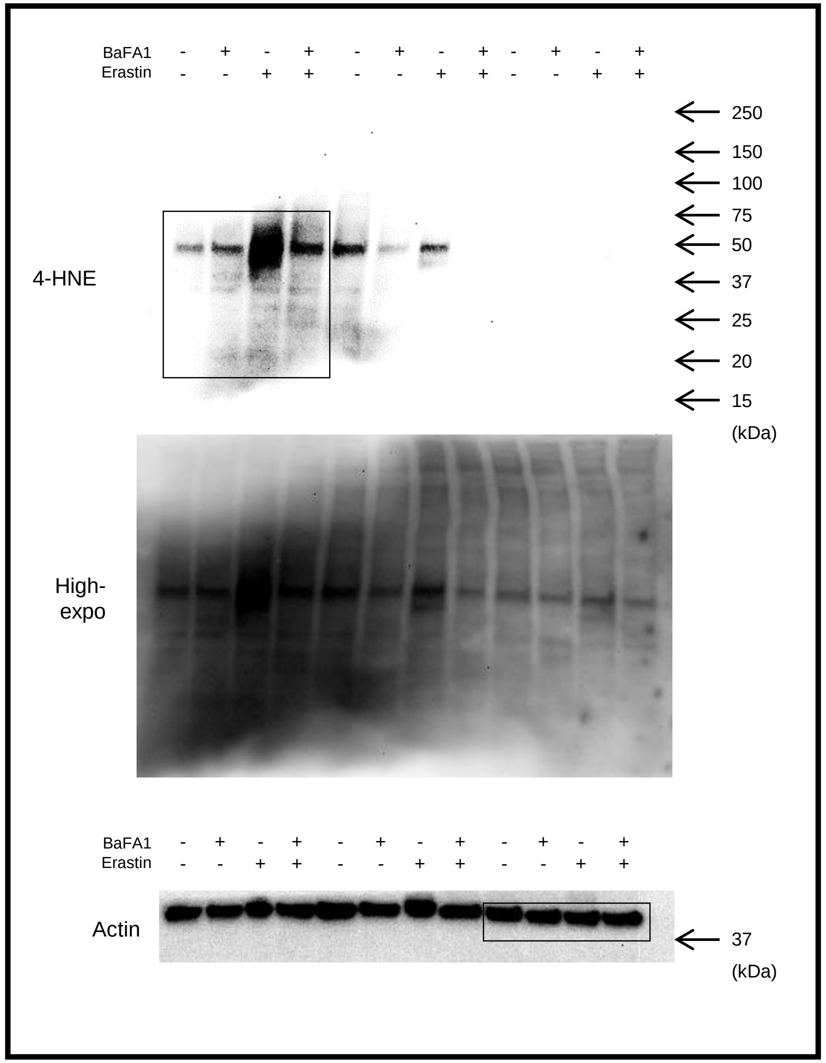

Figure 7G

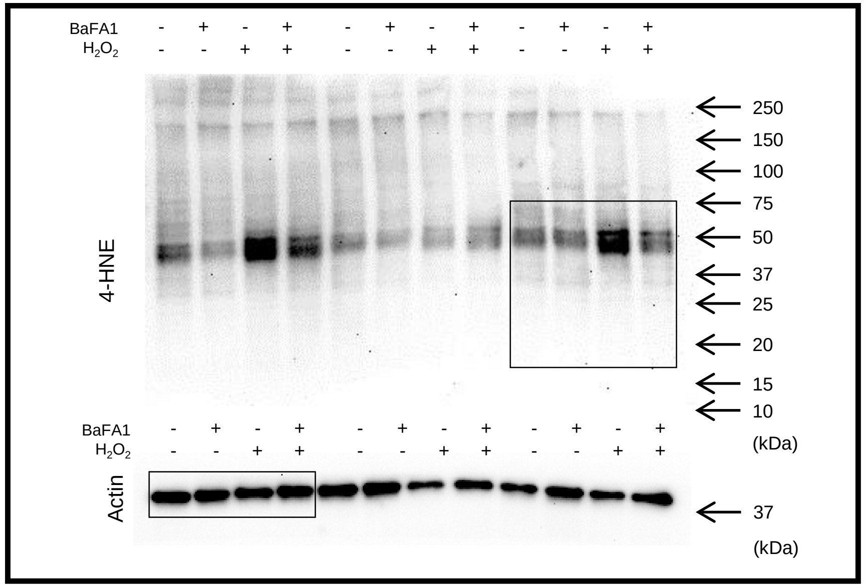

Figure 7-supplemental figure 1A

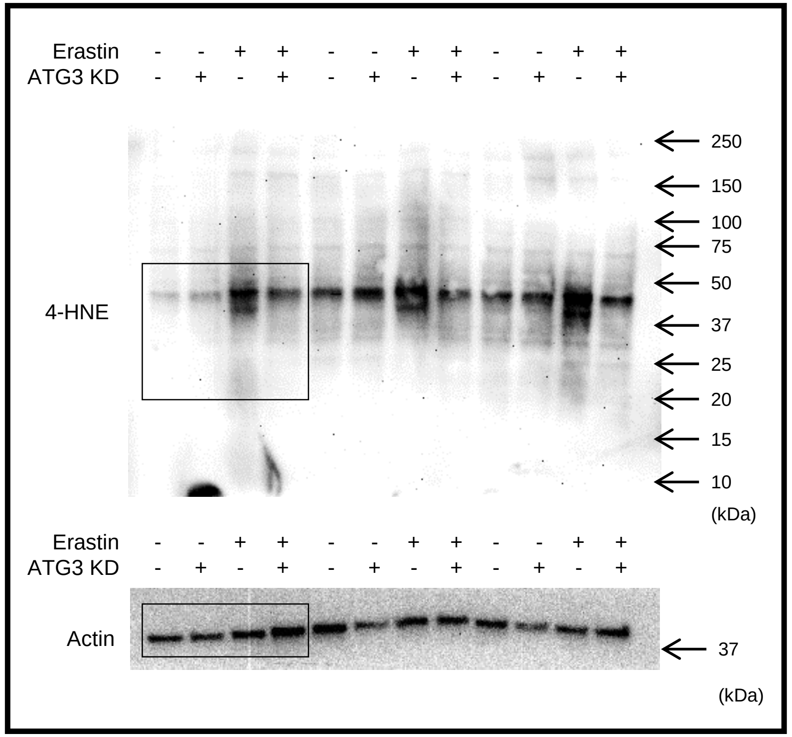

Figure 7-supplemental figure 1C

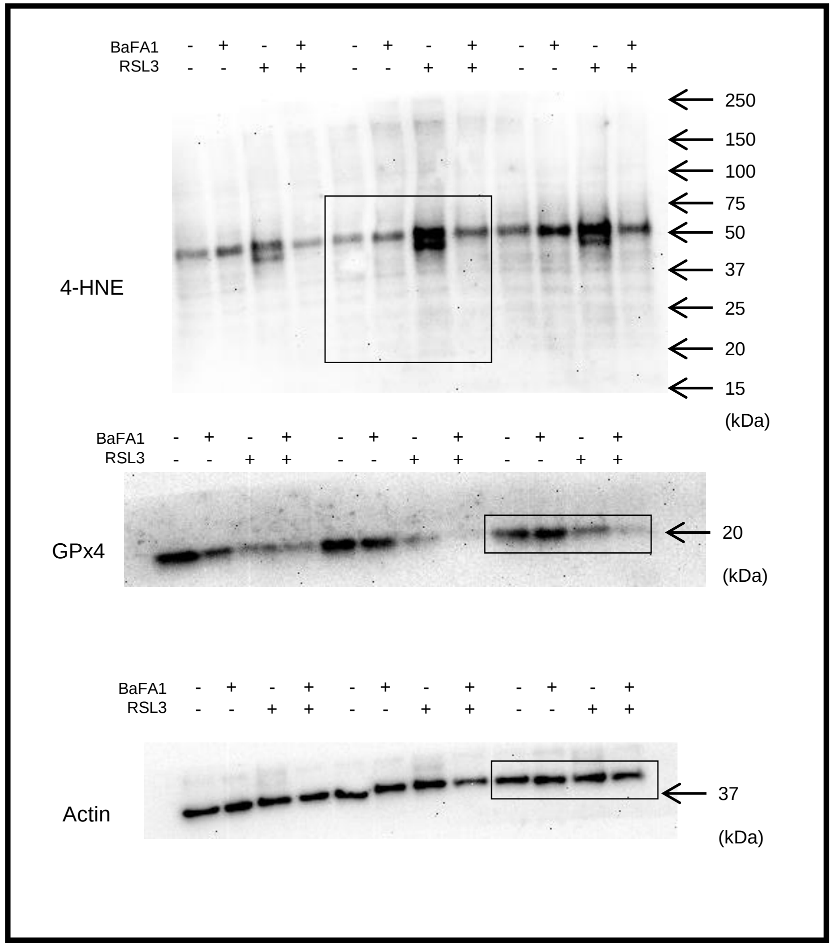

Figure 7-supplemental figure 1F

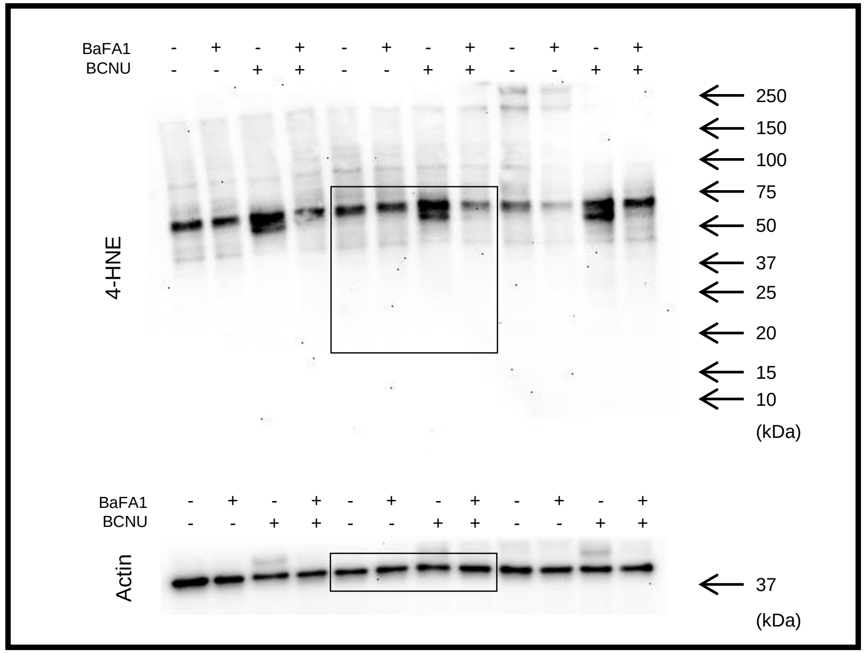

Figure 8C

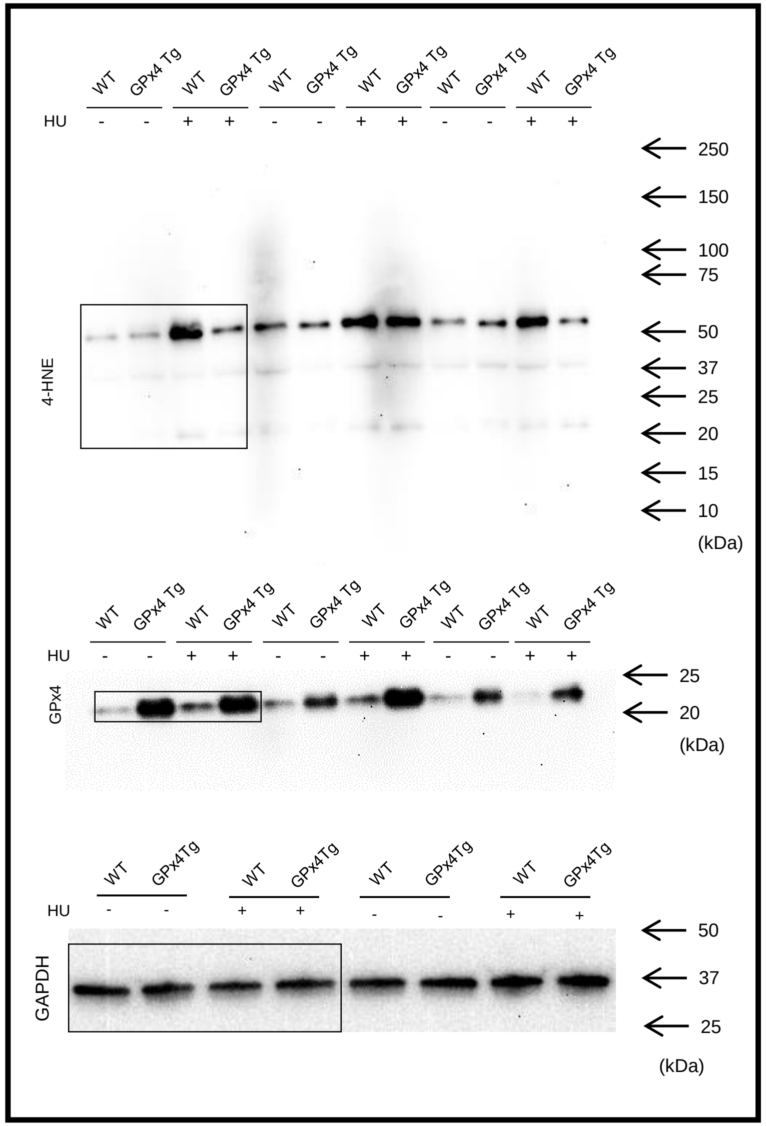

Figure 8F

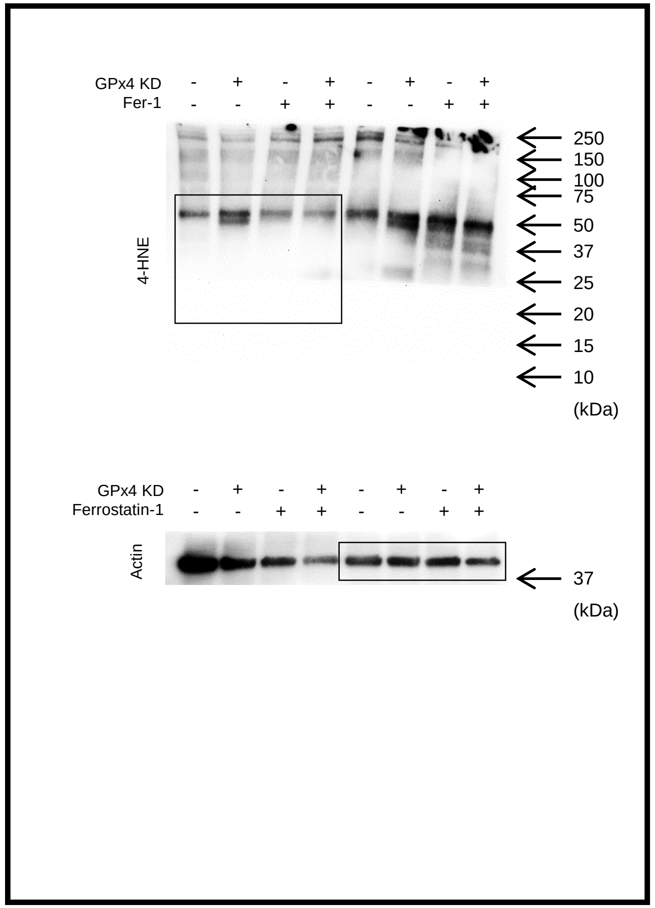

Figure 8-supplemental figure 2D

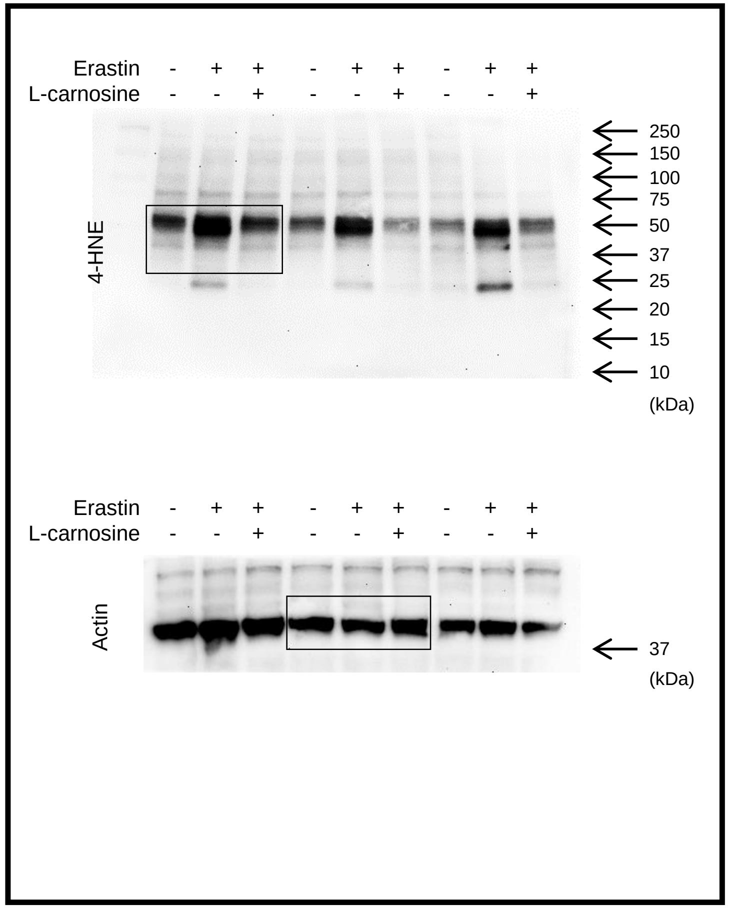

Figure 8-supplemental figure 2G

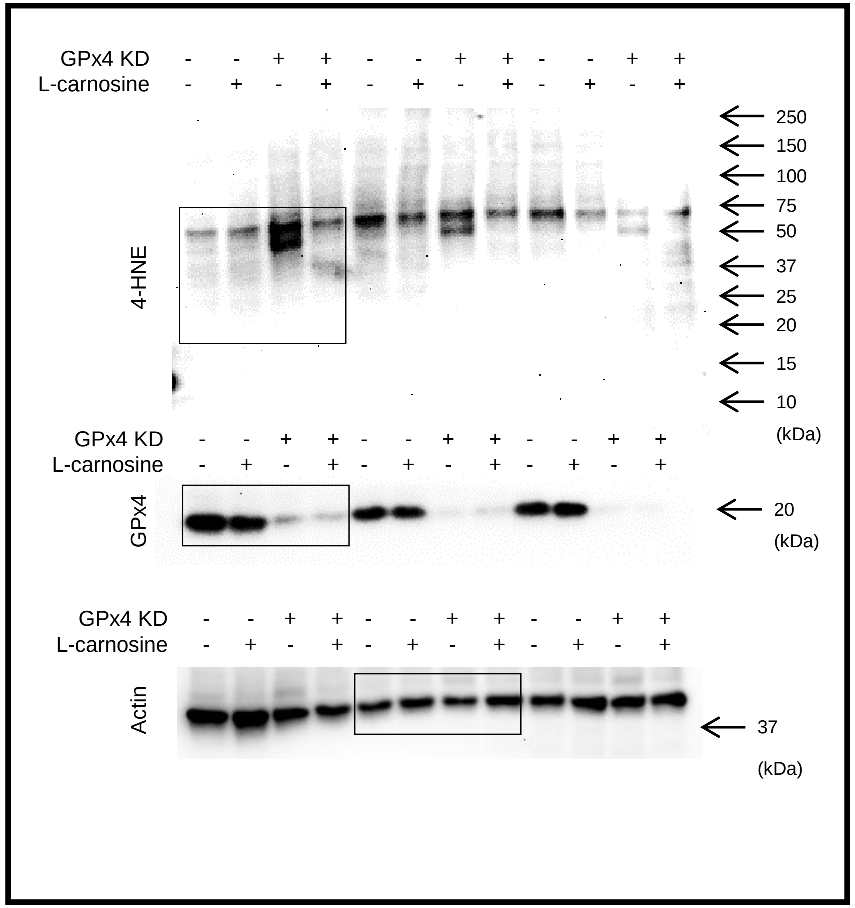

Figure 8-supplemental figure 3A

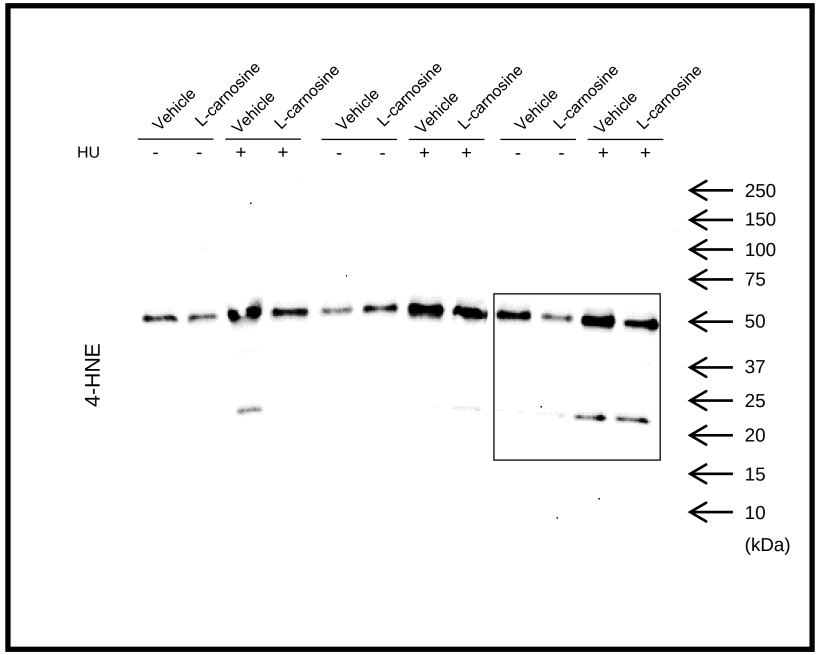

Figure 8-supplemental figure 3E

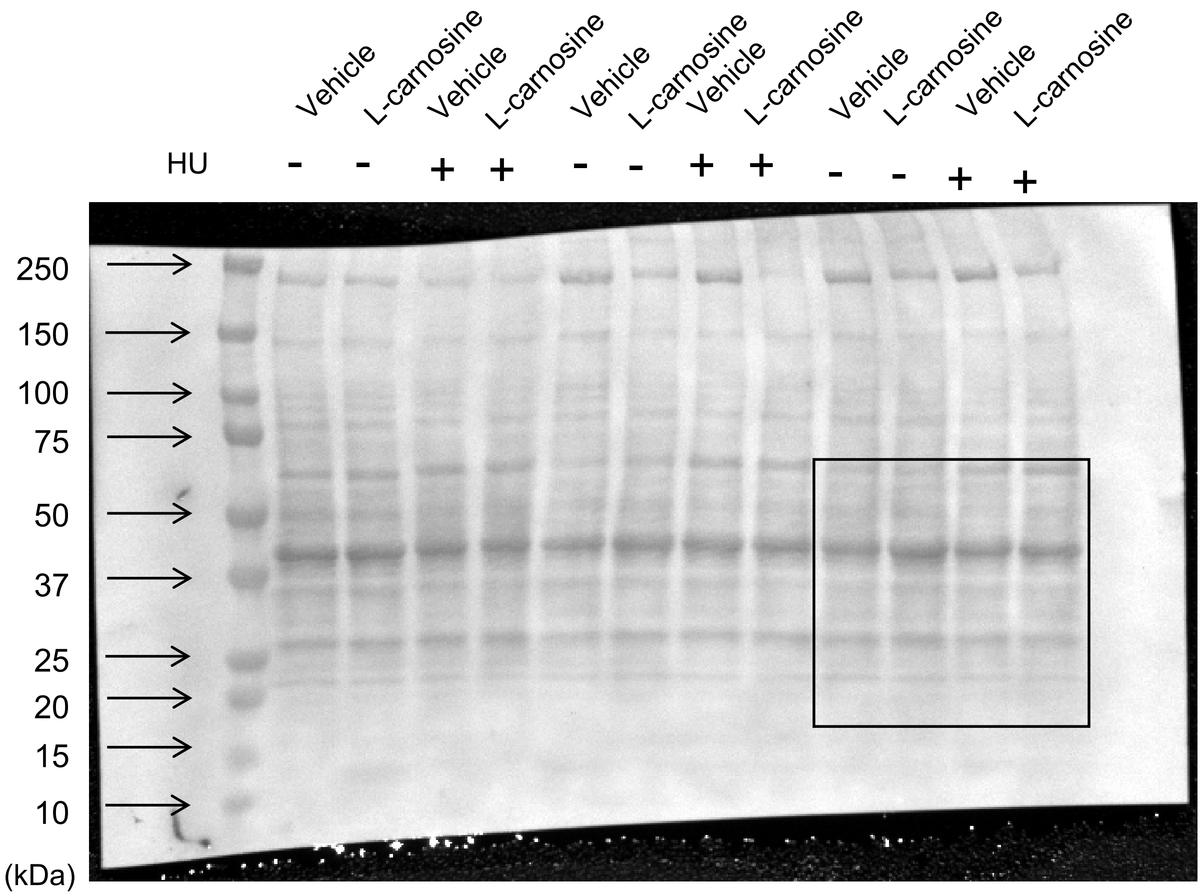

Figure 9B

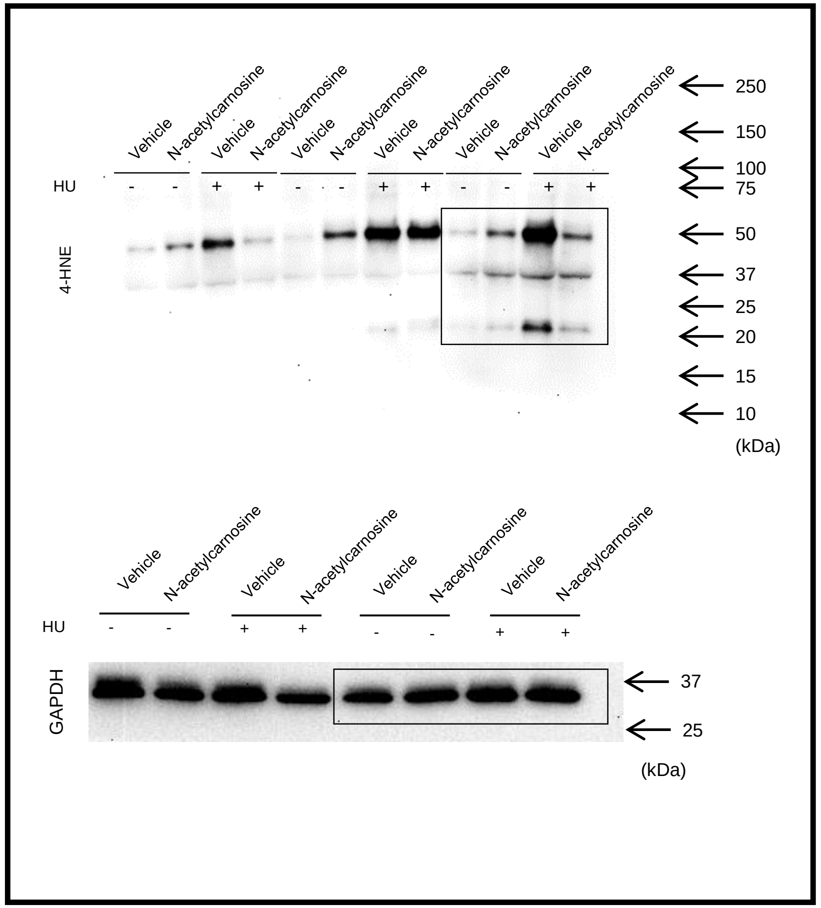
Vehicle
N-acetylcarnosine
Vehicle
N-acetylcarnosine
Vehicle
N-acetylcarnosine
Vehicle
N-acetylcarnosine
Vehicle
N-acetylcarnosine
Vehicle
N-acetylcarnosine
HU
- - + + - - + + - - + +
4-HNE
250
150
100
75
50
37
25
20
15
10
(kDa)
Vehicle
N-acetylcarnosine
Vehicle
N-acetylcarnosine
Vehicle
N-acetylcarnosine
Vehicle
N-acetylcarnosine
HU
- - + + - - + +
GAPDH
37
25
(kDa)

Figure 9-supplemental figure 1A

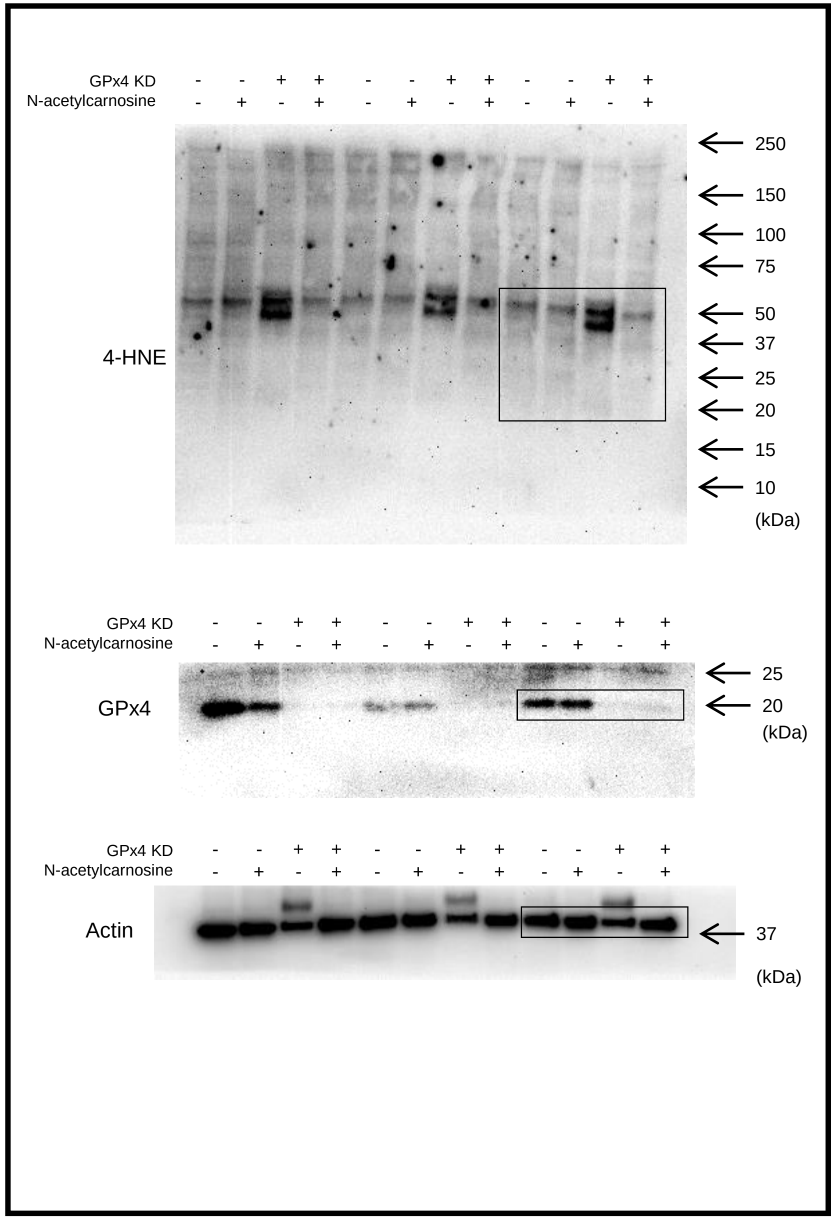

Figure 9-supplemental figure 1D

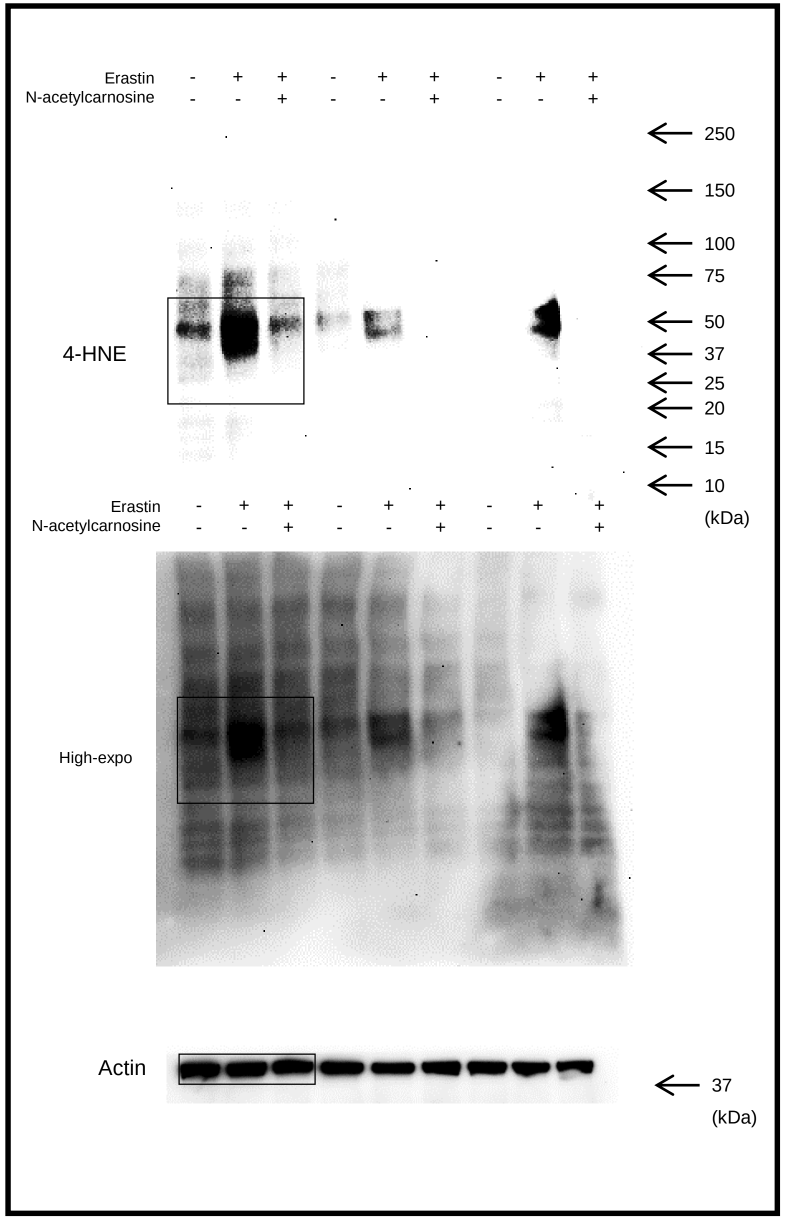

Figure 9-supplemental figure 2F

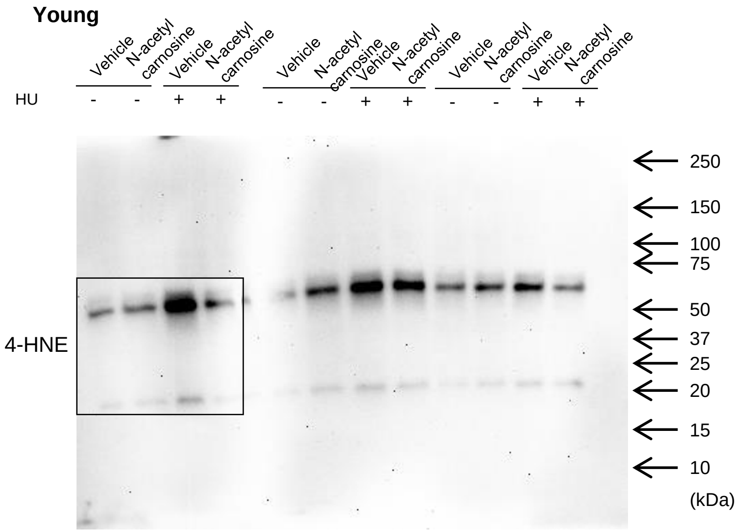

Figure 9-supplemental figure 2F

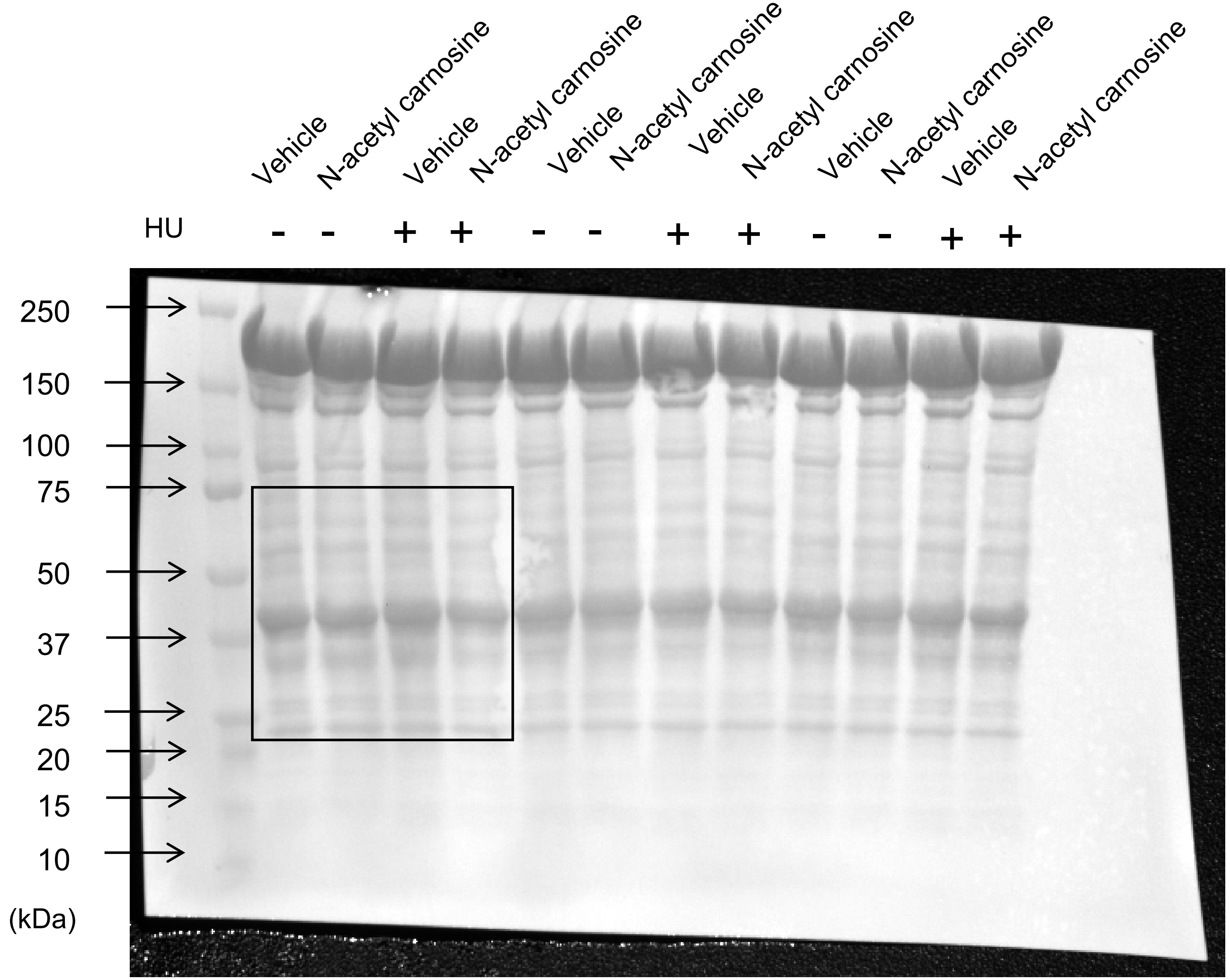

Supplement: Source data 1. [file elife-85289-data1.pdf]
